# On the existence of isoperimetric regions in manifolds with nonnegative Ricci curvature and Euclidean volume growth

**DOI:** 10.1007/s00526-022-02193-9

**Published:** 2022-03-04

**Authors:** Gioacchino Antonelli, Elia Bruè, Mattia Fogagnolo, Marco Pozzetta

**Affiliations:** 1 Scuola Normale Superiore, Piazza dei Cavalieri, 7, 56126 Pisa, Italy; 2 Institute for Advanced Study, 1 Einstein Dr., Princeton, NJ 05840 USA; 3 Centro di Ricerca Matematica Ennio De Giorgi, Scuola Normale Superiore, Piazza dei Cavalieri, 3, 56126 Pisa, Italy; 4 Dipartimento di Matematica e Applicazioni, Università di Napoli Federico II, Via Cintia, Monte S. Angelo, 80126 Napoli, Italy

**Keywords:** 49Q20, 49J45, 53A35, 53C23, 49J40

## Abstract

In this paper we provide new existence results for isoperimetric sets of large volume in Riemannian manifolds with nonnegative Ricci curvature and Euclidean volume growth. We find sufficient conditions for their existence in terms of the geometry at infinity of the manifold. As a byproduct we show that isoperimetric sets of big volume always exist on manifolds with nonnegative sectional curvature and Euclidean volume growth. Our method combines an asymptotic mass decomposition result for minimizing sequences, a sharp isoperimetric inequality on nonsmooth spaces, and the concavity property of the isoperimetric profile. The latter is new in the generality of noncollapsed manifolds with Ricci curvature bounded below.

## Introduction

Given a Riemannian manifold $(M^n,g)$ of dimension $n\ge 2$ we denote by ${\mathsf {d}}$, ${{\,\mathrm{vol}\,}}$, and $\mathrm {Ric}$ the Riemannian distance, the volume measure, and the Ricci tensor of $(M^n,g)$, respectively.

For any $V \in [0,{{\,\mathrm{vol}\,}}(M^n))$, the *isoperimetric problem* consists in studying the minimization problem

1.1 $$\begin{aligned} I_{(M^n,g)}(V):=\inf \{P(\Omega ):\Omega \subset M^n \text {set of finite perimeter with} {{\,\mathrm{vol}\,}}(\Omega )=V\} \, , \end{aligned}$$

where $P(\Omega )$ denotes the perimeter of $\Omega$, see Sect. 2. We shall drop the superscript *n* in $(M^n,g)$ and the subscript $(M^n,g)$ in $I_{(M^n,g)}$ when there is no risk of confusion. The function $I_{(M^n,g)}$ is the so-called *isoperimetric profile* of $(M^n,g)$. Any set of finite perimeter $E\subset M^n$ with ${{\,\mathrm{vol}\,}}(E)=V$ and $P(E)=I_{(M^n,g)}(E)$ is called *isoperimetric set* or *isoperimetric region*.

The problem of the existence of isoperimetric regions in the setting of noncompact manifolds is a hard problem that has seen several important progresses in the last years. Let us just mention some of the most important contributions in the field related to the topics of this work. Major results in the application of a direct method for proving existence of isoperimetric sets in manifolds with lower bounds on the Ricci curvature are contained in [63, 69]. The methods employed there have been generalized in [13]. As we are going to see, such generalization will be fundamental for the present work. In dimension 2, a complete positive answer to the existence issue of isoperimetric sets under nonnegative curvature has been given in [74]. The existence of isoperimetric sets in 3-manifolds with nonnegative scalar curvature and asymptotically flat asymptotics has been established in [25]. Existence results for isoperimetric sets of large volumes in asymptotically flat manifolds were also obtained in [41, 42, 68], in asymptotically hyperbolic spaces in [33], and in the asymptotically conical case in [34]. When the ambient space is a nonnegatively Ricci curved cone, isoperimetric regions exist for any given volume and are actually characterized [66]. In Euclidean solid cones, the problem has been investigated in [76]. In more general convex bodies, it is treated in [57].

Differential properties of the isoperimetric profile, some of which are generalized in the present paper, have been studied in [18–21, 65]. A regularity theory for isoperimetric sets was established for example in [50, 64, 81, 83], and, recently, it has been partly generalized in [14].

### Main existence results

In this paper we provide new existence results for isoperimetric regions of large volume in the setting of manifolds with nonnegative Ricci curvature and *Euclidean volume growth*, i.e., such that

$$\begin{aligned} \mathrm {AVR}(M^n,g):=\lim _{r\rightarrow +\infty }\frac{{{\,\mathrm{vol}\,}}(B_r(x))}{\omega _nr^n} \in (0,1] \, , \end{aligned}$$

where $\omega _n$ is the volume of the *n*-dimensional unit ball. The above limit exists as a consequence of the Bishop–Gromov monotonicity (cf. Theorem 2.11). The same notion can be given for ${{\mathsf {C}}}{{\mathsf {D}}}(0,n)$ metric measure spaces $(X,{\mathsf {d}},{\mathfrak {m}})$ (replacing ${{\,\mathrm{vol}\,}}$ with ${\mathfrak {m}}$), a class of nonsmooth spaces with a synthetic notion of nonnegative Ricci curvature and dimension bounded above by $n \ge 1$, see Sect. 2.2.

The Euclidean volume growth assumption implies that $(M^n,g)$ is *noncollapsed*, i.e. there exists $v>0$ such that ${{\,\mathrm{vol}\,}}(B_1(x))\ge v$ for every $x\in M$. The latter can be shown to be equivalent to $I_{(M^n,g)}(V)\ne 0$ for some (and in fact for any) $V>0$ (cf. Proposition 2.18). Hence noncollapsedness is necessary for the existence of isoperimetric regions (see also the counterexamples discussed in [13, Section 4.3]).

Before stating our first result we recall that $(X,{\mathsf {d}},{\mathfrak {m}})$ is said to *split (a line)* if it is isometric as a metric measure space to $({\mathbb {R}}\times Y, {\mathsf {d}}_{\mathbb {R}}\otimes {\mathsf {d}}_Y, {\mathfrak {m}}_{\mathbb {R}}\otimes {\mathfrak {m}}_Y)$ for some $(Y,{\mathsf {d}}_Y,{\mathfrak {m}}_Y)$. Here ${\mathsf {d}}_{{\mathbb {R}}^k}, {\mathfrak {m}}_{{\mathbb {R}}^k}$ are just the Euclidean distance and the Lebesgue measure on ${\mathbb {R}}^k$, respectively, for any $k\in {\mathbb {N}}$.

#### Theorem 1.1

Let $(M^n,g)$ be a complete noncompact noncollapsed Riemannian manifold with $\mathrm {Ric}\ge 0$. We write

$$\begin{aligned} (M^n,g) = ({\mathbb {R}}^k\times N^{n-k},g_{{\mathbb {R}}^k}+ g_N) , \end{aligned}$$

where $(N^{n-k}, g_N)$ does not split a line, and $0\le k\le n$. Assume that there exists $\varepsilon >0$ such that every pmGH limit $(X_\infty ,{\mathsf {d}}_\infty ,{\mathfrak {m}}_\infty ,x_\infty )$ of sequences $\{(M,{\mathsf {d}},{{\,\mathrm{vol}\,}},p_i)\}_{i\in {\mathbb {N}}_{\ge 0}}$ with $p_i:=(0,x_i)\in M$, and ${\mathsf {d}}_N(x_i,x_0)\rightarrow +\infty$ as $i\rightarrow +\infty$, satisfies

$$\begin{aligned} \mathrm {AVR}(X_\infty ,{\mathsf {d}}_\infty ,{\mathfrak {m}}_\infty )\ge \mathrm {AVR}(M^n,g) + \varepsilon . \end{aligned}$$

Then there exists $V_0>0$ such that for every $V\ge V_0$ there exists an isoperimetric region of volume *V* in *M*.

Recall that any sequence $\{(M,{\mathsf {d}},{{\,\mathrm{vol}\,}},p_i)\}_{i\in {\mathbb {N}}}$ admits limit points in the pmGH topology as a consequence of the Gromov compactness theorem (see Definition 2.4 and Remark 2.7). Such limits could in general be nonsmooth, and just belong to the class of $\mathsf {RCD}$ spaces, that are pointed metric metric measure spaces with a suitable synthetic notion of Ricci curvature bounded below.

The hypothesis in Theorem 1.1 is verified when $\mathrm {AVR}(M^n,g)>0$ and the manifold *N* possesses no asymptotic cones that split a line (see Definition 2.14 for the notion of asymptotic cone). Hence we deduce the following.

#### Theorem 1.2

Let $(M^n,g)$ be a complete Riemannian manifold with $\mathrm {Ric}\ge 0$ and $\mathrm {AVR}(M^n,g)>0$. We write

$$\begin{aligned} (M^n,g) = ({\mathbb {R}}^k\times N^{n-k},g_{{\mathbb {R}}^k}+ g_N) \, , \end{aligned}$$

where $(N^{n-k}, g_N)$ does not split a line, and $0\le k\le n$. If no asymptotic cone of *N* splits a line, then there is $V_0>0$ such that for every $V\ge V_0$ there exists an isoperimetric region of volume *V* in *M*.

It is well-known that when $(M^n,g)$ has nonnegative sectional curvature the asymptotic cone is unique and it splits if and only if the manifold splits, see [16, pages 58-59], [54, Proposition 4.2]. A clear account on the classical method for proving the latter properties is contained in [51]. Since in this work we are interested in the case of Euclidean volume growth, we provide a proof of the previous facts on asymptotic cones in such special case in Theorem 4.6. The latter phenomenon marks an important difference with the nonnegative Ricci curvature, see [36, Theorem 1.4] and Sect. 1.4 below. In turn, we derive the following existence theorem.

#### Theorem 1.3

Let $(M^n,g)$ be a complete Riemannian manifold with nonnegative sectional curvature and Euclidean volume growth. Then there exists $V_0>0$ such that for every $V\ge V_0$ there exists an isoperimetric region of volume *V*.

Let us briefly discuss the condition in Theorem 1.2. When *M* does not split any line, it amounts to say that no asymptotic cone splits a line. It is not hard to see that the latter is equivalent to the following spectral gap property: there exists $\varepsilon >0$ such that

1.2 $$\begin{aligned} \uplambda _1(-\Delta _Z) \ge n-1 + \varepsilon \end{aligned}$$

for all the cross-sections $(Z,{\mathsf {d}}_Z)$ of asymptotic cones. Here $\uplambda _1(-\Delta _Z)$ denotes the first eigenvalue of the Laplacian in *Z*. The same condition has already appeared in [34, Theorem 1], where its validity was guaranteed by the uniqueness and smoothness of the asymptotic cone.

We point out that (1.2) has a clear variational meaning: $-\Delta _Z -(n-1)$ coincides with the Jacobi operator associated to the second variation of the perimeter of $B_1(z)\subset C(Z)$, where $z\in C(Z)$ is the tip point. Hence, (1.2) simply says that $B_1(z)$ is a strictly stable isoperimetric region on *C*(*Z*). In view of this we can, at least at a heuristic level, rephrase our statement as follows: isoperimetric regions of big volume exist provided the isoperimetric sets of the model spaces at infinity are uniformly stable.

### Concavity of the isoperimetric profile

We now state our last result regarding concavity properties of the isoperimetric profile. This will be an important ingredient in the proof of the previous existence theorems and has an independent interest. Given $I\subset {\mathbb {R}}$ an interval, and given $f:I\rightarrow {\mathbb {R}}$, we set, for any $x\in I$,

$$\begin{aligned} {\overline{D}}^2 f(x):=\limsup _{h\rightarrow 0}\frac{f(x+h)+f(x-h)-2f(x)}{h^2}. \end{aligned}$$

#### Theorem 1.4

Suppose that $(M^n,g)$ is noncollapsed and that $\mathrm {Ric}\ge (n-1)K$ for $K \le 0$. Then for any ${\widetilde{V}}\in (0,\mathrm {vol}(M^n))$ there are $\delta >0$ and $C\ge 0$ such that the function $V\mapsto I_{(M^n,g)}(V) - CV^2$ is concave on $({\widetilde{V}}- \delta ,{\widetilde{V}}+ \delta )$. Moreover,

1.3 $$\begin{aligned} {\overline{D}}^2 I_{(M^n,g)}(V)\le \frac{(n-1)|K|}{I_{(M^n,g)}(V)}, \end{aligned}$$

holds for every $V\in (0,\mathrm {vol}(M^n))$, and equivalently the inequality

1.4 $$\begin{aligned} I''_{(M^n,g)}\le \frac{(n-1)|K|}{I_{(M^n,g)}}, \end{aligned}$$

holds in the distributional sense on $(0,\mathrm {vol}(M^n))$. As a consequence, $I_{(M^n,g)}$ is twice differentiable almost everywhere and at any value *V* of twice differentiability it holds the pointwise estimate

1.5 $$\begin{aligned} I_{(M^n,g)}''(V) \le \frac{(n-1)|K|}{I_{(M^n,g)}(V)}. \end{aligned}$$

If $K=0$ then $I_{(M^n,g)}$ is concave on $(0,\mathrm {vol}(M^n))$.

The previous result is obtained under the natural assumptions for the study of the isoperimetric problem, namely noncollapsedness and Ricci bounded below. In particular, differently from the classical results in [18–21, 63, 65], the existence of isoperimetric regions is not assumed *a priori*, and, in fact, we will employ Theorem 1.4 as a tool to provide existence. On the other hand, it appears challenging also relying on Nardulli’s generalized existence of isoperimetric sets as done in [63], since in our setting the mass lost at infinity is recovered in $\mathsf {RCD}$ spaces where the classical GMT tools needed to compute the second variation of the perimeter are not available at present.^1^

To this end, in order to prove Theorem 1.4, we introduce an approximation of the perimeter functional on the manifold *M* by a sequence of penalized perimeters $P_k$ given by the sum of the usual perimeter and a potential term. The penalization in the definition of $P_k$, see (3.1), implies the existence of volume constrained minimizers for $P_k$ for any *k*. Also, the profile $I_k$ corresponding to $P_k$, see (3.2), converges to the isoperimetric profile $I_{(M^n,g)}$ of the manifold locally uniformly. Hence Theorem 1.4 eventually follows by studying the concavity properties of $I_k$ by means of the existence of minimizers for $P_k$, and then by passing the estimates to the limit with respect to *k*.

As a consequence of Theorem 1.4, we get that under the same assumptions the isoperimetric profile is a locally Lipschitz function (Corollary 3.5), which improves the result of local Hölder continuity contained in [67].

As anticipated, Theorem 1.4 has useful consequences for the the existence of isoperimetric regions in manifolds with nonnegative Ricci curvature. This is due to the fact that the concavity of the profile provides information on the asymptotic behavior of $I_{(M^n,g)}$ and $I'_{(M^n,g)}$, see Corollary 3.6. Under the additional assumption of Euclidean volume growth, we can further conclude that $I_{(M^n,g)}$ is strictly increasing (Corollary 3.8). The latter result has to be compared with [75], where the author considers hypotheses on the sectional curvature instead, but without assumptions on the asymptotic volume ratio.

### Strategy of proof of the existence results

Broadly speaking the proof of Theorem 1.1 is based on the direct method of the Calculus of Variations and a concentration-compactness argument, firstly employed in the study of the isoperimetric problem by Nardulli in [69]. Given $V>0$ we consider a minimizing sequence $E_i$ such that ${{\,\mathrm{vol}\,}}(E_i)=V$ and $\lim _{i\rightarrow \infty }P(E_i) = I(V)$, and we aim at studying limits in a generalized sense. Notice that the existence of a limit in $L^1(M^n,{{\,\mathrm{vol}\,}})$ would immediately give the existence of isoperimetric regions of volume *V*, as a consequence of the lower semicontinuity of the perimeter. The main enemy for the existence of the limit is the possibility of having pieces of $E_i$ escaping at infinity.

The asymptotic decomposition result in [13] (see Theorem 2.16 below) tells us that, for noncollapsed Riemannian manifolds with Ricci curvature bounded from below, the mass lost at infinity can be recovered by looking at subsets of metric measure spaces obtained as pmGH limit points of $(M^n,{\mathsf {d}}, {{\,\mathrm{vol}\,}}, p_i)$, where $p_i\rightarrow \infty$ is a suitable sequence. In the setting of Theorem 1.1, we succeed in controlling the isoperimetric profile of these limit spaces, with the aim of showing that escaping at infinity is not “isoperimetrically convenient”. To this aim we employ the recent result [17, Theorem 1.1], showing that the isoperimetric constant on ${{\mathsf {C}}}{{\mathsf {D}}}(0, n)$ spaces is, roughly speaking, realized by balls of infinite radius, and thus that it is explicitly related to the asymptotic volume ratio. This result generalizes earlier analogous inequalities in the smooth setting [1, 22, 44, 53]. Notice that the pmGH limits at infinity of a noncollpased nonnegatively Ricci curved manifold are indeed ${{\mathsf {C}}}{{\mathsf {D}}}(0,n)$ spaces. We show, in particular, that in the setting of Theorem 1.1 it is more convenient for minimizing sequences of large volume not to lose mass at infinity along *N*. On the other hand, if part of the mass of any minimizing sequence is lost at infinity along the factor ${\mathbb {R}}^k$, it can be taken back by means of translations along the Euclidean factor.

To perform such a plan we need to know the exact asymptotic behaviour for large volumes of the isoperimetric profile *I* and its derivative $I'$, for an arbitrary manifold with nonnegative Ricci curvature. The latter piece of information is used to ensure that every minimizing sequence of sets of sufficiently big volumes can lose at most one piece at infinity along *N*, see Lemma 4.1.

Loosely speaking, the behaviour at infinity of *I* is the same as the isoperimetric profile of a cone with opening $\mathrm {AVR}(M^n,g)$. The computation of the asymptotics of *I* is a direct consequence of Bishop–Gromov comparison theorem and the sharp isoperimetric inequality, while the computation of the asymptotics of $I'$ is a byproduct of the concavity property proven in Theorem 1.4.

We finally stress that the extremal case in the hypothesis in Theorem 1.1, i.e., when $\mathrm {AVR}(X_{\infty })=1$, happens when every limit $X_\infty$ is isometric to ${\mathbb {R}}^n$. In this simpler case, since the mass of a minimizing sequence lost along the factor ${\mathbb {R}}^k$ can be taken back as mentioned above, by a minor variation of the proof of [13, Theorem 5.2] one even gets existence of isoperimetric regions for every volume.

Let us comment on the proof of Theorem 1.2. We show that, under the hypotheses of Theorem 1.2, the hypotheses of Theorem 1.1 are satisfied. This is done in two steps, which correspond to item (i) and (ii) of Lemma 4.2. First, if no asymptotic cone of *N* splits a line, then the density of every point at distance 1 from the line of the tips of every asymptotic cone of *M* is uniformly bigger than $\mathrm {AVR}(M^n,g)$. The latter is due to a compactness argument and to the fact that if a point at distance 1 from the line of the tips of an asymptotic cone ${\mathbb {R}}^k\times C$ of *M* has density equal to $\mathrm {AVR}(M^n,g)$, then there is a line in ${\mathbb {R}}^k\times C$ which is not contained in ${\mathbb {R}}^k$ and then, by the splitting theorem, one has a splitting of the cone *C*, which is an asymptotic cone of *N*, resulting in a contradiction. Second, by means of the volume convergence theorem, a lower bound on the density of points at distance 1 from the line of the tips of the asymptotic cones is readily seen to imply a lower bound on the $\mathrm {AVR}$ of the pmGH limits at infinity of the manifold along *N*. It is worth pointing out the reference [32], where this kind of cone splitting argument originated from.

The result in item (i) of Lemma 4.2 discussed above allows to get also other nontrivial existence results for the isoperimetric problem. It can be proved, see Theorem 4.3, that for manifolds as in Theorem 1.2, if every point at distance 1 from the line of the tips of every asymptotic cone is regular, i.e., has density one, then isoperimetric regions exist for every volume. In particular this implies, see Corollary 4.4, that if a Riemannian manifold with nonnegative Ricci curvature and Euclidean volume growth is such that every asymptotic cone has a smooth cross section, then isoperimetric regions exist for every volume. We point out that when the dimension is 2 this partially recovers the aforementioned existence result of isoperimetric sets in nonnegative curvature due to Ritoré [74].

### Sharpness and counterexamples

While the assumptions in Theorem 1.1 turn out to be always satisfied in the setting of manifolds with nonnegative sectional curvature, giving rise to Theorem 1.3, the following example borrowed from [55, pp. 913-914] shows that this is not always the case on manifolds with nonnegative Ricci curvature. Let us consider the metric

$$\begin{aligned} g:=f(r)^2 dt^2 + dr^2 + \eta (r)^2 g_{{\mathbb {S}}^{k-1}} \quad \text {for} (t,r,p) \in {\mathbb {R}}\times [0,+\infty ) \times {\mathbb {S}}^{k-1} \simeq {\mathbb {R}}^{k+1}, k\ge 3 \, , \end{aligned}$$

where

$$\begin{aligned} f(r)= & {} (b + r^2)^{(\beta +2 - k)/2} + c \, \quad \alpha , \beta \in (0,1), \, b, c>1\, ,\, k-\beta > 2 + \alpha \, , \\ \eta (r)= & {} \frac{1}{2}r + \frac{1}{2a} \int _0^r \int _t^\infty \xi (s) d s \, , \end{aligned}$$

for some smooth $\xi :[0, \infty ) \rightarrow [0, \infty )$ satisfying $\xi (t) = t$ on [0, 1], $\xi >0$ on [1, 2] and $\xi (t)=t^{-1-\alpha }$ on $[2, \infty )$. The parameter $a>0$ is chosen such that $\eta '(0) = 1$.

It is possible to prove that *g* has nonnegative Ricci curvature provided *b* and *c* are chosen big enough. It is easy to see that $({\mathbb {R}}^{k+1}, g)$ has Euclidean volume growth and admits a unique asymptotic cone isometric to ${\mathbb {R}}\times C({\mathbb {S}}^{k-1}_\rho )$, where $\rho = 1/2$ is the radius of ${\mathbb {S}}^{k-1}_\rho$, which is the cross section of the cone $C({\mathbb {S}}^{k-1}_\rho )$. Observe that the asymptotic cone splits a line while $({\mathbb {R}}^{k+1}, g)$ does not. Moreover, *g* is invariant under the translation along the direction $\partial _t$. In particular, if $P_i=(t_i,r_i,p_i)$ satisfies $\sup _i r_i <+\infty$ and $|t_i| \rightarrow \infty$, we deduce that $({\mathbb {R}}^{k+1}, {\mathsf {d}}_g, {{\,\mathrm{vol}\,}}_g, P_i) \rightarrow ({\mathbb {R}}^{k+1}, {\mathsf {d}}_g, {{\,\mathrm{vol}\,}}_g, 0)$ in the pmGH topology. Hence the assumptions of Theorem 1.1 are *not* satisfied.

On the other hand, along sequences $Q_i=(t_i,r_i,p_i)$ with $r_i\rightarrow \infty$, one checks that $({\mathbb {R}}^{k+1}, {\mathsf {d}}_g, {{\,\mathrm{vol}\,}}_g, Q_i)$ converges to the Euclidean space $({\mathbb {R}}^{k+1}, {\mathsf {d}}_\mathrm{eu}, {\mathcal {L}}^{k+1}, 0)$ in the pmGH topology. Therefore, by the asymptotic mass decomposition Theorem 2.16, one has that the volume of a minimizing sequence lost at infinity ends up in isoperimetric regions in $({\mathbb {R}}^{k+1},g_\mathrm{eu})$ or in $({\mathbb {R}}^{k+1}, g)$. However, the presence of such limit regions in $({\mathbb {R}}^{k+1},g_\mathrm{eu})$ easily contradicts the minimizing property of the initial sequence (cf. [13, Theorem 5.1 & 5.2] and [65, Theorem 3.5]), while limit isoperimetric regions in $({\mathbb {R}}^{k+1}, g)$ can be obviously brought back into the original $({\mathbb {R}}^{k+1}, g)$, recovering the missing volume. Therefore we obtain that $({\mathbb {R}}^{k+1}, g)$ has isoperimetric regions for *any* volume.

All in all, we conclude that our assumptions in Theorem 1.1 are not necessary for the existence of isoperimetric sets in the setting of manifolds with nonnegative Ricci curvature.

To date we do not know whether manifolds with nonnegative Ricci curvature and Euclidean volume growth always admit isoperimetric regions for big volumes, and we plan to investigate further this problem in the future.

### Plan of the paper

In Sect. 2 we introduce the preliminary material. We briefly describe the class of ${{\mathsf {C}}}{{\mathsf {D}}}$ and $\mathsf {RCD}$ spaces, we recall the notion of pmGH convergence and the mass decomposition theorem (Theorem 2.16), and we prove the mentioned sharp Sobolev inequality (Theorem 2.21). Section 3 is devoted to the proof of Theorem 1.4. In Sect. 4 we prove the existence results Theorem 1.1, Theorem 1.2, Theorem 1.3, and further existence results (Sect. 4.3).

## Definitions and auxiliary results

### Preliminaries on Riemannian manifolds and metric measure spaces

In this preliminary section we introduce basic facts and notations about Riemannian manifolds and metric measure spaces.

Given two Riemannian manifolds $(M^m,g), (N^n,h)$, we denote with $(M^m\times N^n,g+h)$ the product of the two Riemannian structure. Given a set $E\subset M$, we denote with $\mathrm {vol}(E)$ its *n*-dimensional Hausdorff measure ${\mathcal {H}}^n (E)$ with respect to the distance induced by *g*. This notion coincides with its classical Riemannian volume measure induced by *g*. For the notions of BV and Sobolev spaces on Riemannian manifolds we refer the reader to [61, Section 1]. For every finite perimeter set *E* in $\Omega$ we denote with $P(E,\Omega )$ the perimeter of *E* inside $\Omega$. When $\Omega =M^n$ we simply write *P*(*E*). We denote with ${\mathcal {H}}^{n-1}$ the $(n-1)$-dimensional Hausdorff measure on $M^n$ relative to the distance induced by *g*. If *E* is a $C^1$-hypersurface, *P*(*E*) coincides with its classical area measure induced by *g*. We recall that for every finite perimeter set *E* one has $P(E)={\mathcal {H}}^{n-1}(\partial ^*E)$ and the characteristic function $\chi _E$ belongs to $BV_\mathrm{loc}(M^n, {{\,\mathrm{vol}\,}})$ with generalized gradient $D\chi _E = \nu _E {\mathcal {H}}^{n-1}\llcorner \partial ^* E$ for a vector field $\nu _E:M\rightarrow T M^n$ defined $|D\chi _E|$-a.e. with $|\nu _E|=1$ at $|D\chi _E|$-a.e. point, where $\partial ^* E$ is the essential boundary of *E* and $|D\chi _E|$ is the total variation of the measure $D\chi _E$.

We recall the following terminology.

#### Definition 2.1

(*Convergence of finite perimeter sets*) Let $(M^n,g)$ be a Riemannian manifold. We say that a sequence of measurable (with respect to the volume measure) sets $E_i$ *locally converges* to a measurable set *E* if the characteristic functions $\chi _{E_i}$ converge to $\chi _E$ in $L^1_\mathrm{loc}(M^n,g)$. In such a case we simply write that $E_i\rightarrow E$ locally on $M^n$.

If the sets $E_i$ have also locally finite perimeter, that is, $P(E_i,\Omega )<+\infty$ for any *i* and any bounded open set $\Omega$, we say that $E_i\rightarrow E$ *in the sense of finite perimeter sets* if $E_i\rightarrow E$ locally on $M^n$ and the sequence of measures $D\chi _{E_i}$ locally weakly* converges as measures, that is, with respect to the duality with compactly supported continuous functions. In such a case, *E* has locally finite perimeter and the weak* limit of $D\chi _{E_i}$ is $D\chi _E$.

#### Remark 2.2

(*Approximation of finite perimeter sets with smooth sets*) It can be proved, see [67, Lemma 2.3], that when $M^n$ is a complete Riemannian manifold every finite perimeter set $\Omega$ with $0<{{\,\mathrm{vol}\,}}(\Omega )<+\infty$ and ${{\,\mathrm{vol}\,}}(\Omega ^c)>0$ is approximated by relatively compact sets $\Omega _i$ in $M^n$ with smooth boundary such that ${{\,\mathrm{vol}\,}}(\Omega _i)={{\,\mathrm{vol}\,}}(\Omega )$ for every $i\in {\mathbb {N}}$, ${{\,\mathrm{vol}\,}}(\Omega _i\Delta \Omega )\rightarrow 0$ when $i\rightarrow +\infty$, and $P(\Omega _i)\rightarrow P(\Omega )$ when $i\rightarrow +\infty$. Thus, by approximation, one can deduce that

$$\begin{aligned} I(V)=\inf \{{\mathcal {H}}^{n-1}(\partial \Omega ):\Omega \Subset M^n \text {has smooth boundary}, {{\,\mathrm{vol}\,}}(\Omega )=V\}, \end{aligned}$$

see [67, Theorem 1.1].

We also need to recall the definition of the simply connected radial models with constant sectional curvature.

#### Definition 2.3

(*Models of constant sectional curvature, cf.* [72, Example 1.4.6]) Let us define

$$\begin{aligned} {{\,\mathrm{sn}\,}}_K(r) := {\left\{ \begin{array}{ll} (-K)^{-\frac{1}{2}} \sinh ((-K)^{\frac{1}{2}} r) &{} K<0,\\ r &{} K=0,\\ K^{-\frac{1}{2}} \sin (K^{\frac{1}{2}} r) &{} K>0. \end{array}\right. } \end{aligned}$$

If $K>0$, then $((0,\pi /\sqrt{K}]\times {\mathbb {S}}^{n-1},\mathrm dr^2+\mathrm {sn}_K^2(r)g_1)$, where $g_1$ is the canonical metric on ${\mathbb {S}}^{n-1}$, is the radial model of dimension *n* and constant sectional curvature *K*. The metric can be smoothly extended at $r=0$, and thus we shall write that the the metric is defined on the ball ${\mathbb {B}}^n_{\pi /\sqrt{K}} \subset {\mathbb {R}}^n$. The Riemannian manifold $({\mathbb {B}}^n_{\pi /\sqrt{K}}, g_K:=\mathrm dr^2+\mathrm {sn}_K^2(r)g_1)$ is the unique (up to isometry) simply connected Riemannian manifold of dimension *n* and constant sectional curvature $K>0$.

If instead $K\le 0$, then $((0,+\infty )\times {\mathbb {S}}^{n-1},\mathrm dr^2+\mathrm {sn}_K^2(r)g_1)$ is the radial model of dimension *n* and constant sectional curvature *K*. Extending the metric at $r=0$ analogously yields the unique (up to isometry) simply connected Riemannian manifold of dimension *n* and constant sectional curvature $K\le 0$, in this case denoted by $({\mathbb {R}}^n,g_K)$.

We denote by *v*(*n*, *K*, *r*) the volume of the ball of radius *r* in the (unique) simply connected Riemannian manifold of sectional curvature *K* of dimension *n*, and by *s*(*n*, *K*, *r*) the volume of the boundary of such a ball. In particular $s(n,K,r)=n\omega _n\mathrm {sn}_K^{n-1}(r)$ and $v(n,K,r)=\int _0^rn\omega _n\mathrm {sn}_K^{n-1}(r)\,\mathrm dr$, where $\omega _n$ is the Euclidean volume of the Euclidean unit ball in ${\mathbb {R}}^n$.

On ${\mathbb {R}}^n$ we denote with ${\mathsf {d}}_{{\mathbb {R}}^n}$, $g_{{\mathbb {R}}^n}$, and ${\mathfrak {m}}_{{\mathbb {R}}^n}$, respectively, the Euclidean distance, the Euclidean metric, and the Lebesgue measure.

Let us now briefly recall the main concepts we will need from the theory of metric measure spaces. We recall that a *metric measure space,* $\mathrm {m.m.s.}$ *for short,* $(X,{\mathsf {d}}_X,{\mathfrak {m}}_X)$ is a triple where $(X,{\mathsf {d}}_X)$ is a locally compact separable metric space and ${\mathfrak {m}}_X$ is a Borel measure bounded on bounded sets. A *pointed metric measure space* is a quadruple $(X,{\mathsf {d}}_X,{\mathfrak {m}}_X,x)$ where $(X,{\mathsf {d}}_X,{\mathfrak {m}}_X)$ is a metric measure space and $x\in X$ is a point.

For simplicity, and since it will always be our case, we will always assume that given $(X,{\mathsf {d}}_X,{\mathfrak {m}}_X)$ a m.m.s. the support $\mathrm{spt}\,{\mathfrak {m}}_X$ of the measure ${\mathfrak {m}}_X$ is the whole *X*.

Given two m.m.s. $(X,{\mathsf {d}}_X,{\mathfrak {m}}_X)$ and $(Y,{\mathsf {d}}_Y,{\mathfrak {m}}_Y)$, we denote by $(X\times Y,{\mathsf {d}}_X\otimes {\mathsf {d}}_Y,{\mathfrak {m}}_X\otimes {\mathfrak {m}}_Y)$ the product m.m.s., where

$$\begin{aligned} {\mathsf {d}}_X\otimes {\mathsf {d}}_Y((x,y),(x',y')):=\sqrt{{\mathsf {d}}_X(x,x')^2+{\mathsf {d}}_Y(y,y')^2}, \qquad \forall x,x'\in X,\quad \forall y,y'\in Y, \end{aligned}$$

and ${\mathfrak {m}}_X\otimes {\mathfrak {m}}_Y$ is the usual product of measures.

We assume the reader to be familiar with the notion of pointed measured Gromov–Hausdorff convergence, referring to [82, Chapter 27] and to [24, Chapter 7 and 8] for an overview on the subject. In the following treatment we introduce the pmGH-convergence already in a proper realization even if this is not the general definition. Nevertheless, the (simplified) definition of Gromov–Hausdorff convergence via a realization is equivalent to the standard definition of pmGH convergence in our setting, because in the applications we will always deal with locally uniformly doubling measures, see [49, Theorem 3.15 and Section 3.5]. The following definition is taken from the introductory exposition of [4].

#### Definition 2.4

(*pGH and pmGH convergence*) A sequence $\{ (X_i, {\mathsf {d}}_i, x_i) \}_{i\in {\mathbb {N}}}$ of pointed metric spaces is said to converge in the *pointed Gromov–Hausdorff topology, in the* $\mathrm {pGH}$ *sense for short,* to a pointed metric space $(Y, {\mathsf {d}}_Y, y)$ if there exist a complete separable metric space $(Z, {\mathsf {d}}_Z)$ and isometric embeddings

$$\begin{aligned} \begin{aligned}&\Psi _i:(X_i, {\mathsf {d}}_i) \rightarrow (Z,{\mathsf {d}}_Z), \qquad \forall \, i\in {\mathbb {N}},\\&\Psi \, \, :(Y, {\mathsf {d}}_Y) \, \rightarrow (Z,{\mathsf {d}}_Z), \end{aligned} \end{aligned}$$

such that for any $\varepsilon ,R>0$ there is $i_0(\varepsilon ,R)\in {\mathbb {N}}$ such that

$$\begin{aligned} \Psi _i(B_R^{X_i}(x_i)) \subset \left[ \Psi (B_R^Y(y))\right] _\varepsilon , \qquad \Psi (B_R^{Y}(y)) \subset \left[ \Psi _i(B_R^{X_i}(x_i))\right] _\varepsilon , \end{aligned}$$

for any $i\ge i_0$, where $[A]_\varepsilon :=\{ z\in Z \ :\ {\mathsf {d}}_Z(z,A)\le \varepsilon \}$ for any $A \subset Z$.

Let ${\mathfrak {m}}_i$ and $\mu$ be given in such a way $(X_i,{\mathsf {d}}_i,{\mathfrak {m}}_i,x_i)$ and $(Y,{\mathsf {d}}_Y,\mu ,y)$ are m.m.s. If in addition to the previous requirements we also have $(\Psi _i)_\sharp {\mathfrak {m}}_i \rightharpoonup \Psi _\sharp \mu$ with respect to duality with continuous bounded functions on *Z* with bounded support, then the convergence is said to hold in the *pointed measured Gromov–Hausdorff topology, or in the* $\mathrm {pmGH}$ *sense for short*.

We remark that in the setting we will deal with, product structures are stable under pmGH convergence, i.e., if $(X_n,{\mathsf {d}}_{X_n},{\mathfrak {m}}_{X_n},x_n)\rightarrow (X,{\mathsf {d}}_X,{\mathfrak {m}}_X,x)$ and $(Y_n,{\mathsf {d}}_{Y_n},{\mathfrak {m}}_{Y_n},y_n)\rightarrow (Y,{\mathsf {d}}_Y,{\mathfrak {m}}_Y,y)$ in the pmGH sense then

$$\begin{aligned} (X_n\times Y_n,{\mathsf {d}}_{X_n}\otimes {\mathsf {d}}_{Y_n},{\mathfrak {m}}_{X_n}\otimes {\mathfrak {m}}_{Y_n}, (x_n,y_n))\rightarrow (X\times Y,{\mathsf {d}}_X\otimes {\mathsf {d}}_Y,{\mathfrak {m}}_X\otimes {\mathfrak {m}}_Y,(x,y)), \end{aligned}$$

in pmGH.

### $\mathsf {RCD}$ spaces

Since we will use part of the $\mathsf {RCD}$ theory just as an instrument for our purposes and since we will never use in the paper the specific definition of $\mathsf {RCD}$ space, we just outline the main references on the subject and we refer the interested reader to the survey of Ambrosio [3] and the references therein.

After the introduction, in the independent works [79, 80] and [58], of the curvature dimension condition ${{\mathsf {C}}}{{\mathsf {D}}}(K,n)$ encoding in a synthetic way the notion of Ricci curvature bounded from below by *K* and dimension bounded above by *n*, the definition of $\mathsf {RCD}(K,n)$ m.m.s. was first proposed in [48] and then studied in [11, 43, 47], see also [27] for the equivalence between the $\mathsf {RCD}^*(K,n)$ and the $\mathsf {RCD}(K,n)$ condition. The infinite dimensional counterpart of this notion had been previously investigated in [9], see also [8] for the case of $\sigma$-finite reference measures.

#### Remark 2.5

(*pmGH limit of RCD spaces*) We recall that, whenever it exists, a pmGH limit of a sequence $\{(X_i,{\mathsf {d}}_i,{\mathfrak {m}}_i,x_i)\}_{i\in {\mathbb {N}}}$ of (pointed) $\mathsf {RCD}(K,n)$ spaces is still an $\mathsf {RCD}(K,n)$ metric measure space.

Due to the compatibility of the $\mathsf {RCD}$ condition with the smooth case of Riemannian manifolds with Ricci curvature bounded from below and to its stability with respect to pointed measured Gromov–Hausdorff convergence, limits of smooth Riemannian manifolds with Ricci curvature uniformly bounded from below by *K* and dimension uniformly bounded from above by *n* are $\mathsf {RCD}(K,n)$ spaces. Then the class of $\mathsf {RCD}$ spaces includes the class of Ricci limit spaces, i.e., limits of sequences of Riemannian manifolds with the same dimension and with Ricci curvature uniformly bounded from below [28–31]. An extension of noncollapsed Ricci limit spaces is the class of $\mathsf {RCD}(K,n)$ space where the reference measure is the *n*-dimensional Hausdorff measure relative to the distance, introduced and studied in [12, 39, 56]. As a consequence of the rectifiability of $\mathsf {RCD}$ spaces [62] (see also [23] for an alternative proof), we remark that if $(X,{\mathsf {d}},{\mathcal {H}}^n)$ is an $\mathsf {RCD}(K,n)$ space then *n* is an integer.

We state the volume convergence theorems obtained by Gigli and De Philippis in [39, Theorem 1.2 and Theorem 1.3], which are the synthetic version of the celebrated volume convergence of Colding [35] (see also [28]).

#### Theorem 2.6

Let $\{(X_i,{\mathsf {d}}_i,{\mathcal {H}}^n,x_i)\}_{i\in {\mathbb {N}}}$ be a sequence of pointed $\mathsf {RCD}(K,n)$ m.m.s. with $K\in {\mathbb {R}}$ and $n\in [1,+\infty )$. Assume that $(X_i,{\mathsf {d}}_i,x_i)$ converges in the pGH topology to $(X,{\mathsf {d}},x)$. Then precisely one of the following happens

- $\limsup _{i\rightarrow \infty }{\mathcal {H}}^n\left( B_1(x_i)\right) >0$. Then the $\limsup$ is a limit, it coincides with the volume of the limit unitary ball and $(X_i,{\mathsf {d}}_i,{\mathcal {H}}^n,x_i)$ converges in the pmGH topology to $(X,{\mathsf {d}},{\mathcal {H}}^n,x)$. Hence $(X,{\mathsf {d}},{\mathcal {H}}^n)$ is an $\mathsf {RCD}(K,n)$ m.m.s. endowed with the *n*-dimensional Hausdorff measure;
- $\lim _{i\rightarrow \infty }{\mathcal {H}}^n(B_1(x_i))=0$. In this case we have $\dim _{H}(X,{\mathsf {d}})\le n-1$, where we denoted by $\dim _H(X,{\mathsf {d}})$ the Hausdorff dimension of $(X,{\mathsf {d}})$.

Moreover, for $K\in {\mathbb {R}}$ and $n\in [1,+\infty )$, let ${\mathbb {B}}_{K,n,R}$ be the collection of all equivalence classes up to isometry of closed balls of radius *R* in $\mathsf {RCD}(K,n)$ spaces, equipped with the Gromov-Hausdorff distance. Then the map ${\mathbb {B}}_{K,n,R}\ni Z\rightarrow {\mathcal {H}}^n(Z)$ is real-valued and continuous.

#### Remark 2.7

(*Gromov precompactness theorem for RCD spaces*) Here we recall the synthetic variant of Gromov’s precompactness theorem for $\mathsf {RCD}$ spaces, see [39, Equation (2.1)]. Let $\{(X_i,{\mathsf {d}}_i,{\mathfrak {m}}_i,x_i)\}_{i\in {\mathbb {N}}}$ be a sequence of $\mathsf {RCD}(K_i,n)$ spaces with $n\in [1,+\infty )$, ${{\,\mathrm{spt}\,}}({\mathfrak {m}}_i)=X_i$ for every $i\in {\mathbb {N}}$, ${\mathfrak {m}}_i(B_1(x_i))\in [v,v^{-1}]$ for some $v\in (0,1)$ and for every $i\in {\mathbb {N}}$, and $K_i\rightarrow K\in {\mathbb {R}}$. Then there exists a subsequence pmGH-converging to some $\mathsf {RCD}(K,n)$ space $(X,{\mathsf {d}},{\mathfrak {m}},x)$ with ${{\,\mathrm{spt}\,}}({\mathfrak {m}})=X$.

We conclude this part by recalling a few basic definitions and results concerning the perimeter functional in the setting of metric measure spaces (see [2, 6, 60]).

#### Definition 2.8

(*BV* *functions and perimeter on m.m.s.*) Let $(X,{\mathsf {d}},{\mathfrak {m}})$ be a metric measure space. A function $f \in L^1(X,{\mathfrak {m}})$ is said to belong to the space of *bounded variation functions* $BV(X,{\mathsf {d}},{\mathfrak {m}})$ if there is a sequence $f_i \in \mathrm{Lip}_{\mathrm {loc}}(X)$ such that $f_i \rightarrow f$ in $L^1(X,{\mathfrak {m}})$ and $\limsup _i \int _X \mathrm{lip \,}f_i \,\mathrm d{\mathfrak {m}}< +\infty$, where $\mathrm{lip \,}u (x) :=\limsup _{y\rightarrow x} \frac{|u(y)-u(x)|}{{\mathsf {d}}(x,y)}$ is the *slope* of *u* at *x*, for any accumulation point $x\in X$, and $\mathrm{lip \,}u(x):=0$ if $x\in X$ is isolated. In such a case we define

$$\begin{aligned} |Df|(A) :=\inf \left\{ \liminf _i \int _A \mathrm{lip \,}f_i \,\mathrm d{\mathfrak {m}}\ :\ f_i \in \mathrm{Lip}_\mathrm{loc}(A), f_i \rightarrow f \text {in} L^1(A,{\mathfrak {m}}) \right\} , \end{aligned}$$

for any open set $A\subset X$.

If $E\subset X$ is a Borel set and $A\subset X$ is open, we define the *perimeter* *P*(*E*, *A*) *of* *E* *in* *A* by

$$\begin{aligned} P(E,A) :=\inf \left\{ \liminf _i \int _A \mathrm{lip \,}u_i \,\mathrm d{\mathfrak {m}}\ :\ u_i \in \mathrm{Lip}_\mathrm{loc}(A), u_i \rightarrow \chi _E \text {in} L^1_\mathrm{loc}(A,{\mathfrak {m}}) \right\} , \end{aligned}$$

We say that *E* has *finite perimeter* if $P(E,X)<+\infty$, and we denote by $P(E):=P(E,X)$. Let us remark that the set functions $|Df|, P(E,\cdot )$ above are restrictions to open sets of Borel measures that we denote by $|Df|, |D\chi _E|$ respectively, see [6], and [60].

The *isoperimetric profile of* $(X,{\mathsf {d}},{\mathfrak {m}})$ is

$$\begin{aligned} I_{X}(V):=\inf \left\{ P(E) \ :\ E\subset X \text {Borel}, {\mathfrak {m}}(E)=V \right\} , \end{aligned}$$

for any $V\in [0,{\mathfrak {m}}(X))$. If $E\subset X$ is Borel with ${\mathfrak {m}}(E)=V$ and $P(E)=I_X(V)$, then we say that *E* is an *isoperimetric region*.

It follows from classical approximation results (cf. Remark 2.2) that the above definition yields the usual notion of perimeter on any Riemannian manifold $(M^n,g)$ recalled at the beginning of this section.

We will need the following standard approximation result, that can be obtained by combining [6, Theorem 1.1] with [40, Theorem 4.5.3 and Proposition 4.5.6].

#### Lemma 2.9

Let $(X,{\mathsf {d}})$ be a complete and separable metric space, and let ${\mathfrak {m}}$ be a nonnegative measure, finite on bounded sets. Then, for any $f\in BV(X,{\mathsf {d}},{\mathfrak {m}})$ there exists a sequence $(f_k)\subset \mathrm{Lip}(X,{\mathsf {d}})$, where $f_k$ has bounded support for any *k*, such that $f_k \rightarrow f$ pointwise ${\mathfrak {m}}$-a.e. and in $L^1(X,{\mathfrak {m}})$, and $\int _X \mathrm{lip \,}(f_k) d {\mathfrak {m}}\rightarrow |Df|(X)$ as $k\rightarrow \infty$.

The following general coarea formula will be employed in obtaining the sharp Sobolev inequality on ${{\mathsf {C}}}{{\mathsf {D}}}(0, n)$ spaces below.

#### Remark 2.10

(*Coarea formula on metric measure spaces*) Let $(X,{\mathsf {d}},{\mathfrak {m}})$ be a metric measure space. Let us observe that from the definitions given above, a Borel set *E* with finite measure has finite perimeter if and only if the characteristic function $\chi _E$ belongs to $BV(X,{\mathsf {d}},{\mathfrak {m}})$.

If $f \in BV(X,{\mathsf {d}},{\mathfrak {m}})$, then $\{f>\alpha \}$ has finite perimeter for a.e. $\alpha \in {\mathbb {R}}$ and the *coarea formula* holds

$$\begin{aligned} \int _X u \,\mathrm d|Df| = \int _{-\infty }^{+\infty } \left( \int _X u \,\mathrm d\left|D\chi _{\{f>\alpha \}}\right| \right) \,\mathrm d\alpha , \end{aligned}$$

for any Borel function $u:X\rightarrow [0,+\infty ]$, see [60, Proposition 4.2]. If *f* is also continuous and nonnegative, then $|Df|(\{f=\alpha \})=0$ for *every* $\alpha \in [0,+\infty )$ and the *localized coarea formula* holds

$$\begin{aligned} \int _{\{a<f<b\}} u \,\mathrm d|Df| = \int _a^b \left( \int _X u \,\mathrm d\left|D\chi _{\{f>\alpha \}}\right| \right) \,\mathrm d\alpha , \end{aligned}$$

for every Borel function $u:X\rightarrow [0,+\infty ]$ and every $0\le a< b < +\infty$, see [4, Corollary 1.9].

We recall a statement for the classical Bishop–Gromov volume and perimeter comparison. The conclusions (2.1), (2.2), and the rigidity part of Theorem 2.11 are consequences, e.g., of [45, Theorem 3.101], [77, Theorem 1.2 and Theorem 1.3], and the arguments within their proofs. The conclusion (2.3) follows from [73, Corollary 2.22, item (i)] and the coarea formula.

#### Theorem 2.11

(Bishop–Gromov comparison) Let $(M^n,g)$ be a complete Riemannian manifold such that $\mathrm {Ric}\ge (n-1)K$ on $M^n$ for some $K \in {\mathbb {R}}$. Let us set $T_K:=+\infty$ if $K\le 0$, and $T_K:=\pi /\sqrt{K}$ if $K>0$. Then, for every $p\in M$ and for $r \le T_K$ the following hold

2.1 $$\begin{aligned}&\frac{{{\,\mathrm{vol}\,}}(B_r(p))}{v(n,K,r)}\rightarrow 1\,\text {as }\, r\rightarrow 0\, \text {and it is nonincreasing}, \end{aligned}$$

2.2 $$\begin{aligned}&\frac{P(B_r(p))}{s(n, K, r)}\rightarrow 1 \,\text {as }\, r\rightarrow 0\, \text {and it is almost everywhere nonincreasing}, \end{aligned}$$

2.3 $$\begin{aligned}&\frac{P(B_r(p))}{s(n, K, r)} \le \frac{{{\,\mathrm{vol}\,}}(B_r(p))}{v(n,K,r)}. \end{aligned}$$

#### Remark 2.12

(Bishop–Gromov comparison theorem on m.m.s.) Let us recall that for an arbitrary ${{\mathsf {C}}}{{\mathsf {D}}}((n-1)K,n)$ space $(X,{\mathsf {d}},{\mathfrak {m}})$ the classical Bishop–Gromov volume comparison (cf. Theorem 2.11) still holds. More precisely, for a fixed $x\in X$, the function ${\mathfrak {m}}(B_r(x))/v(n,K,r)$ is nonincreasing in *r* and the function $P(B_r(x))/s(n,K,r)$ is essentially nonincreasing in *r*, i.e., $P(B_R(x))/s(n,K,R) \le P(B_r(x))/s(n,K,r)$ for almost every radii $R\ge r$, see [82, Theorem 18.8, Equation (18.8), Proof of Theorem 30.11]. Moreover, it holds that $P(B_r(p))/s(n, K, r)\le {{\,\mathrm{vol}\,}}(B_r(p))/v(n,K,r)$ for any $r>0$, indeed the last inequality follows from the monotonicity of the volume and perimeter ratios together with the coarea formula on balls.

Moreover, if $(X,{\mathsf {d}},{\mathcal {H}}^n)$ is an $\mathsf {RCD}((n-1)K,n)$ space, one can conclude that ${\mathcal {H}}^n$-almost every point has a unique measure Gromov–Hausdorff tangent isometric to ${\mathbb {R}}^n$ ( [39, Theorem 1.12]), and thus, from the volume convergence in Theorem 2.6, we get

2.4 $$\begin{aligned} \lim _{r\rightarrow 0}\frac{{\mathcal {H}}^n(B_r(x))}{v(n,K,r)}=\lim _{r\rightarrow 0}\frac{{\mathcal {H}}^n(B_r(x))}{\omega _nr^n}=1, \qquad \text {for}\, {\mathcal {H}}^n \text {-almost every} x, \end{aligned}$$

where $\omega _n$ is the volume of the unit ball in ${\mathbb {R}}^n$. Moreover, since the density function $x\mapsto \lim _{r\rightarrow 0}{\mathcal {H}}^n(B_r(x))/\omega _nr^n$ is lower semicontinuous ( [39, Lemma 2.2]), the latter (2.4) implies that the density is bounded above by the constant 1. Hence, from the monotonicity at the beginning of the remark we deduce that, if $(X,{\mathsf {d}},{\mathcal {H}}^n)$ is an $\mathsf {RCD}((n-1)K,n)$ space, then for every $x\in X$ we have ${\mathcal {H}}^n(B_r(x))\le v(n,K,r)$ for every $r>0$.

Let us also recall a classical definition for the convenience of the reader.

#### Definition 2.13

($\mathrm {AVR}$ *and Euclidean volume growth*) Let $(X,{\mathsf {d}},{\mathfrak {m}})$ be an arbitrary ${{\mathsf {C}}}{{\mathsf {D}}}(0,n)$ space with $n \in [1,+\infty )$. From Remark 2.12, the Bishop–Gromov monotonicity holds, and thus we can define, for an arbitrary $x\in X$,

$$\begin{aligned} \mathrm {AVR}(X,{\mathsf {d}},{\mathfrak {m}}):=\lim _{r\rightarrow +\infty }\frac{{\mathfrak {m}}(B_r(x))}{\omega _nr^n}, \end{aligned}$$

the *asymptotic volume ratio* of $(X,{\mathsf {d}},{\mathfrak {m}})$, where $\omega _n$ is the measure of the unit ball in ${\mathbb {R}}^n$. The previous limit is independent on $x\in M$. Notice that ${\mathfrak {m}}(B_r(x))\ge \mathrm {AVR}(X,{\mathsf {d}},{\mathfrak {m}}) \omega _nr^n$ for every $r>0$, and every $x\in X$. If $\mathrm {AVR}(X,{\mathsf {d}},{\mathfrak {m}})>0$ we say that $(X,{\mathsf {d}},{\mathfrak {m}})$ has *Euclidean volume growth*.

Let us recall the basic definition of asymptotic cone in the setting of $\mathsf {RCD}$ spaces with Euclidean volume growth.

#### Definition 2.14

(Asymptotic cones) Let $(X,{\mathsf {d}},{\mathfrak {m}},x)$ be a pointed $\mathsf {RCD}(0,n)$ space with $\mathrm {AVR}(X,{\mathsf {d}},{\mathfrak {m}})>0$. For every sequence $\{r_i\}_{i\in {\mathbb {N}}}$ with $r_i\rightarrow +\infty$ the sequence of pointed metric measure spaces $\{(X,r_i^{-1}{\mathsf {d}},r_i^{-n}{\mathfrak {m}},x)\}_{i\in {\mathbb {N}}}$ is precompact in the pmGH topology due to Remark 2.7. Every pmGH limit of such a sequence is a metric cone, by a slight modification of the proof of [39, Proposition 2.8] (see also [28, Theorem 7.6]). Any such limit is called an *asymptotic cone of X* and will be denoted by *C* or by *C*(*Z*) when we want to higlight the fact that *Z* is the metric space that is the basis of the cone. We stress that every such *C* is an $\mathsf {RCD}(0,N)$ space.

We refer to [39, Definition 2.7] for the precise definition of metric cone. Notice that the class of asymptotic cones of *X* is independent on the base point $x\in X$. Moreover, it is well known that asymptotic cones may be not unique [28, 36, 71] and may have a nonsmooth cross-section.

### Finite perimeter sets and minimizing sequences for the isoperimetric problem

In this section we recall the main generalized existence result of [13], which will be crucially used in this paper. We need to recall a generalized notion $L^1$-convergence of convergence for sets defined on a sequence of metric measure spaces converging in the pmGH sense. Such a definition is given in [4, Definition 3.1], and it is investigated in [4] capitalizing on the results in [10].

#### Definition 2.15

($L^1$-*strong and* $L^1_{\mathrm {loc}}$ *convergence*) Let $\{ (X_i, {\mathsf {d}}_i, {\mathfrak {m}}_i, x_i) \}_{i\in {\mathbb {N}}}$ be a sequence of pointed metric measure spaces converging in the pmGH sense to a pointed metric measure space $(Y, {\mathsf {d}}_Y, \mu , y)$ and let $(Z,{\mathsf {d}}_Z)$ be a realization as in Definition 2.4.

We say that a sequence of Borel sets $E_i\subset X_i$ such that ${\mathfrak {m}}_i(E_i) < +\infty$ for any $i \in {\mathbb {N}}$ converges *in the* $L^1$ *-strong sense* to a Borel set $F\subset Y$ with $\mu (F) < +\infty$ if ${\mathfrak {m}}_i(E_i) \rightarrow \mu (F)$ and $\chi _{E_i}{\mathfrak {m}}_i \rightharpoonup \chi _F\mu$ with respect to the duality with continuous bounded functions with bounded support on *Z*.

We say that a sequence of Borel sets $E_i\subset X_i$ converges *in the* $L^1_{\mathrm {loc}}$ *-sense* to a Borel set $F\subset Y$ if $E_i\cap B_R(x_i)$ converges to $F\cap B_R(y)$ in $L^1$-strong for every $R>0$.

Observe that in the above definition it makes sense to speak about the convergence $\chi _{E_i}{\mathfrak {m}}_i \rightharpoonup \chi _F\mu$ with respect to the duality with continuous bounded functions with bounded support on *Z* as $(X_i,{\mathsf {d}}_i),(Y,{\mathsf {d}}_Y)$ can be assumed to be topological subspaces of $(Z,{\mathsf {d}}_Z)$ by means of the isometries $\Psi _i,\Psi$ of Definition 2.4, and the measures ${\mathfrak {m}}_i,\mu$ can be then identified with the push-forwards $(\Psi _i)_\sharp {\mathfrak {m}}_i,\Psi _\sharp \mu$ respectively.

Let us recall here for the reader’s convenience the main result of [13], namely [13, Theorem 1.1]. We will crucially need this result for the proof of our main results.

#### Theorem 2.16

(Asymptotic mass decomposition [13, Theorem 1.1]) Let $(M^n,g)$ be a noncollapsed noncompact complete manifold with infinite volume, such that $\mathrm {Ric}\ge K$ for some $K\in (-\infty , 0]$, and let $V>0$. For every minimizing (for the perimeter) sequence of sets $\Omega _i\subset M^n$ of volume *V*, with $\Omega _i$ bounded for any *i*, up to passing to a subsequence, there exist an increasing sequence $\{N_i\}_{i\in {\mathbb {N}}}\subseteq {\mathbb {N}}$, disjoint finite perimeter sets $\Omega _i^c, \Omega _{i,j}^d \subset \Omega _i$, and points $p_{i,j}$, with $1\le j\le N_i$ for any *i*, such that

(i) $\lim _{i} {\mathsf {d}}(p_{i,j},p_{i,\ell }) = \lim _{i} {\mathsf {d}}(p_{i,j},o)=+\infty$, for any $j\ne \ell <L+1$ and any $o\in M^n$, where $L:=\lim _i N_i \in {\mathbb {N}}\cup \{+\infty \}$;
(ii) $\Omega _i^c$ converges to $\Omega \subset M^n$ in the sense of finite perimeter sets (Definition 2.1), and we have ${{\,\mathrm{vol}\,}}(\Omega _i^c)\rightarrow _i {{\,\mathrm{vol}\,}}(\Omega )$, and $P( \Omega _i^c) \rightarrow _i P(\Omega )$. Moreover $\Omega$ is a bounded isoperimetric region on *M*;
(iii) for every $j<L+1$, $(M^n,{\mathsf {d}},{{\,\mathrm{vol}\,}},p_{i,j})$ converges in the pmGH sense to a pointed $\mathsf {RCD}(K,n)$ space $(X_j,{\mathfrak {m}}_j,p_j)$, where ${\mathfrak {m}}_j$ is the *n*-dimensional Hausdorff measure on $(X_j, d_j)$. Moreover there are isoperimetric regions $Z_j \subset X_j$ such that $\Omega ^d_{i,j}\rightarrow _i Z_j$ in $L^1$-strong (Definition 2.15) and $P(\Omega ^d_{i,j}) \rightarrow _i P_{X_j}(Z_j)$;
(iv) it holds that
  2.5 $$\begin{aligned} I_{(M^n,g)}(V) = P(\Omega ) + \sum _{j=1}^{{L}} P_{X_j} (Z_j), \qquad \qquad V={{\,\mathrm{vol}\,}}(\Omega ) + \sum _{j=1}^{{L}} {\mathfrak {m}}_j(Z_j). \end{aligned}$$

We remark that item (ii) in Theorem 2.16 is, in fact, proved in [76, Theorem 2.1], and it consists in the starting point for the rest of the proof of Theorem 2.16.

We will need the following result on the boundedness of isoperimetric regions.

#### Proposition 2.17

( [13, Corollary 4.2]) Let $(M^n,g)$ be a complete noncollapsed Riemannian manifold with $\mathrm {Ric}\ge K$ for some $K \in (- \infty , 0]$. Then the isoperimetric regions of $(M^n,g)$ are bounded.

### The collapsed case

As already observed in [13, Remark 4.7], as a nontrivial consequence of Theorem 2.16 we have that complete noncollapsed manifolds with a lower bound on the Ricci curvature have strictly positive isoperimetric profile for any volume.

With an argument partly inspired by the proof of [57, Proposition 3.13], we are now going to show that, in the nonnegative Ricci case, collapsedness occurs if and only if the isoperimetric profile vanishes for any volume. In particular, no isoperimetric sets exist in this situation.

#### Proposition 2.18

Let $(M^n,g)$ be a complete noncompact Riemannian manifold with $\mathrm {Ric}\ge 0$. Then, $\inf _{x \in M} {{\,\mathrm{vol}\,}}({B_1(x)}) = 0$ if and only if $I(V) = 0$ for any positive volume *V*.

#### Proof

As observed above, if *M* is noncollapsed, then $I(V)>0$ for any *V* by [13, Remark 4.7]. Then let us assume that $\inf _{x \in M} {{\,\mathrm{vol}\,}}(B_1(x))=0$. Observe first that $\inf _{x \in M} {{\,\mathrm{vol}\,}}(B_R(x)) = 0$ for any $R > 0$. Indeed, let $\{x_j\}_{j \in {\mathbb {N}}}$ be a sequence of points such that $\lim _{j \rightarrow \infty } {{\,\mathrm{vol}\,}}B_1(x_j) = 0$. Now, for $R \le 1$ we have ${{\,\mathrm{vol}\,}}B_R(x_j) \le {{\,\mathrm{vol}\,}}(B_1(x_j))\rightarrow _j 0$, while for $R>1$, by the Bishop–Gromov volume comparison, we have ${{\,\mathrm{vol}\,}}(B_R(x_j)) \le R^n {{\,\mathrm{vol}\,}}(B_1(x_j))\rightarrow _j 0$. Fix now a volume $V > 0$, and consider an arbitrary ball $B_R(x)$ such that ${{\,\mathrm{vol}\,}}(B_R(x)) > V$. This is possible since $M^n$ has infinite volume. Since, as we just showed, ${{\,\mathrm{vol}\,}}(B_R(x_j)) \rightarrow 0$ along the sequence $x_j$ above we can in particular find a point ${\overline{x}} \in M$ such that ${{\,\mathrm{vol}\,}}(B_R({\overline{x}})) = V$. By (2.3), we have $P(B_R({\overline{x}})) \le n R^{-1} {{\,\mathrm{vol}\,}}(B_R({\overline{x}}))$, and thus

2.6 $$\begin{aligned} I(V) \le P(B_R({\overline{x}}))\le \frac{n}{R} V. \end{aligned}$$

Observing that the argument actually holds true for arbitrarily big radii *R*, our claim is proved by letting $R \rightarrow \infty$ in (2.6). $\square$

Explicit examples of collapsed manifolds with nonnegative Ricci curvature have been constructed in dimension $n \ge 4$ by Croke and Karcher in [37, Example 1 and Example 2]. Hence for such examples no isoperimetric regions of positive volume can exist.

In the same paper, it is shown that the latter examples do not exist in dimension $n = 2$. As a consequence of Proposition 2.18 and a former result of Ritoré [74, Theorem 1.1], asserting that surfaces with nonnegative curvature admit isoperimetric regions of any given volume, we are able to fully recover such a positive result proved in [37, Theorem A]. We remark that the proof given here is completely different from the one given in [37] and recovers the geometric conclusion of the statement passing through the isoperimetric problem.

#### Corollary 2.19

Let $(M^2,g)$ be a noncompact complete Riemannian surface with $\mathrm {Sect}\ge 0$. Hence there exists $C:=C(M) > 0$ such that

$$\begin{aligned} {{\,\mathrm{vol}\,}}(B(x,1))\ge C, \qquad \forall x\in M. \end{aligned}$$

#### Proof

If the conclusion does not hold, from Proposition 2.18 we get that $I_{(M^2,g)}(V)=0$ for every $V>0$ and hence no isoperimetric regions of positive volume can exist on $(M^2,g)$. But this is in contradiction with [74, Theorem 1.1 & Theorem 2.1]. $\square$

#### Remark 2.20

The proof of the previous result Proposition 2.18 builds on the Bishop–Gromov monotonicity results, which hold for ${{\mathsf {C}}}{{\mathsf {D}}}(0,n)$ spaces, cf. Remark 2.12. In fact, one can show with the same argument that also on a ${{\mathsf {C}}}{{\mathsf {D}}}(0,n)$ space $(X,{\mathsf {d}},{\mathfrak {m}})$, if $\inf _{x\in X}{\mathfrak {m}}(B_1(x))=0$, then the isoperimetric profile identically vanishes.

### Sharp Sobolev inequality on CD(0, n) spaces

Here, we provide a version of the sharp isoperimetric inequality in $\mathrm {CD}(0, n)$ spaces recently obtained in [17] (see also the earlier [1, 22, 44, 53]). Namely, an application of [7] allows to obtain it in terms of the perimeter, in place of the Minkowski content. Moreover, we are not requiring the boundedness of the sets involved. We write all the details, for the readers’ sake. We point out also [70, Proposition 2.4] and the proof of [26, Theorem 3.2], that allow to reach for the same conclusion.

#### Theorem 2.21

(Sharp Sobolev inequality on CD(0,n) spaces) Let $(X,{\mathsf {d}},{\mathfrak {m}})$ be a ${{\mathsf {C}}}{{\mathsf {D}}}(0,n)$ space. Then for any $f\in \text {BV}(X,{\mathsf {d}},{\mathfrak {m}})$ it holds

2.7 $$\begin{aligned} n \omega _n^{\frac{1}{n}} \mathrm {AVR}(X,{\mathsf {d}},{\mathfrak {m}})^{\frac{1}{n}} \left( \int _X |f|^{\frac{n}{n-1}} \,\mathrm d{\mathfrak {m}}\right) ^{\frac{n-1}{n}} \le |D f|(X) \, . \end{aligned}$$

In particular, for any set of finite perimeter *E*, with ${\mathfrak {m}}(E)<\infty$, we have

2.8 $$\begin{aligned} P_X(E)\ge n\omega _n^{\frac{1}{n}}\mathrm {AVR}(X,{\mathsf {d}},{\mathfrak {m}})^{\frac{1}{n}}{\mathfrak {m}}(E)^{\frac{n-1}{n}}. \end{aligned}$$

The isoperimetric inequality [17, Theorem 1.1] reads, for any bounded subset $E\subset X$ of a ${{\mathsf {C}}}{{\mathsf {D}}}(0,n)$ space $(X,{\mathsf {d}},{\mathfrak {m}})$,

2.9 $$\begin{aligned} {\mathfrak {m}}^+(E) \ge n\omega _n^{\frac{1}{n}} \mathrm {AVR}(X,{\mathsf {d}},{\mathfrak {m}})^{\frac{1}{n}} {\mathfrak {m}}(E)^{\frac{n-1}{n}} \, , \end{aligned}$$

where ${\mathfrak {m}}^+$ denotes the (lower) Minkowski content

2.10 $$\begin{aligned} {\mathfrak {m}}^+(E):= \liminf _{r\rightarrow 0} \frac{{\mathfrak {m}}(B_r(E))-{\mathfrak {m}}(E)}{r}\, . \end{aligned}$$

Here we denoted by $B_r(E)$ the *r*-enlargement of *E*, i.e. the set of those points $x\in X$ such that ${\mathsf {d}}(x,y)<r$ for some $y\in E$. The proof of Theorem 2.21 substantially follows from (2.9) together with a relaxation argument [7]. Observe that (2.8) also allows for unbounded sets.

We will need the following elementary lemma proven in [5, Lemma A.24].

#### Lemma 2.22

Let $G:[0,\infty )\rightarrow [0,\infty )$ a nonincreasing measurable function. Then for any $\alpha \ge 1$ we have

2.11 $$\begin{aligned} \alpha \int _0^\infty t^{\alpha -1} G(t) \,\mathrm dt \le \left( \int _0^{\infty } G^{1/\alpha }(t) \,\mathrm dt \right) ^{\alpha } \, . \end{aligned}$$

#### Proof of Theorem 2.21

Let $f:X\rightarrow [0,\infty )$ be a Lipschitz function with bounded support. From [7, Proposition 4.2] we know that

$$\begin{aligned} \int _X \mathrm{lip \,}(f) \,\mathrm d{\mathfrak {m}}= \int _0^{\infty } {\mathfrak {m}}^+(\{f>t\}) \,\mathrm dt, \end{aligned}$$

hence, from (2.9) we deduce

$$\begin{aligned} \int _X \mathrm{lip \,}(f) \,\mathrm d{\mathfrak {m}}\, = \int _0^\infty {\mathfrak {m}}^+(\{f>t\}) \,\mathrm dt \, \ge n \omega _n^{\frac{1}{n}} \mathrm {AVR}(X,{\mathsf {d}},{\mathfrak {m}})^{\frac{1}{n}} \int _0^\infty {\mathfrak {m}}(\{f>t\})^{\frac{n-1}{n}} \,\mathrm dt. \end{aligned}$$

A simple application of Lemma 2.22 coupled with the coarea formula in Remark 2.10 gives

$$\begin{aligned} \int _0^\infty {\mathfrak {m}}(\{f>t\})^{\frac{n-1}{n}} \,\mathrm dt \ge \left( \int _X f^{\frac{n}{n-1}} \,\mathrm d{\mathfrak {m}}\right) ^{\frac{n-1}{n}}\, , \end{aligned}$$

for any nonnegative Lipschitz function with bounded support. We can easily drop the assumption on the sign of *f* by using the decomposition in positive and negative part. Let us finally show (2.7) via approximation argument. Let us fix $f\in BV(X,{\mathsf {d}},{\mathfrak {m}})$, and approximate it with $(f_k)$ as in Lemma 2.9. For any $k\in {\mathbb {N}}$ it holds

$$\begin{aligned} \int _X \mathrm{lip \,}(f_k) \,\mathrm d{\mathfrak {m}}\ge n \omega _n^{\frac{1}{n}} \mathrm {AVR}(X,{\mathsf {d}},{\mathfrak {m}})^{\frac{1}{n}} \left( \int _X |f_k|^{\frac{n}{n-1}} \,\mathrm d{\mathfrak {m}}\right) ^{\frac{n-1}{n}} \, , \end{aligned}$$

and passing to the limit as $k\rightarrow \infty$ we deduce

$$\begin{aligned} |Df|(X)&\ge \liminf _{k\rightarrow \infty } n \omega _n^{\frac{1}{n}} \mathrm {AVR}(X,{\mathsf {d}},{\mathfrak {m}})^{\frac{1}{n}} \left( \int _X |f_k|^{\frac{n}{n-1}} \,\mathrm d{\mathfrak {m}}\right) ^{\frac{n-1}{n}} \\&\ge \omega _n^{\frac{1}{n}} \mathrm {AVR}(X,{\mathsf {d}},{\mathfrak {m}})^{\frac{1}{n}} \left( \int _X |f|^{\frac{n}{n-1}} \,\mathrm d{\mathfrak {m}}\right) ^{\frac{n-1}{n}} \, \end{aligned}$$

where the last inequality follows from Fatou’s lemma.

The last conclusion (2.8) comes by plugging $f=\chi _E$ in (2.7). $\square$

## Concavity properties of the isoperimetric profile

In this section we study concavity properties of the isoperimetric profile of a noncollapsed Riemannian manifold with Ricci bounded from below. We then apply the obtained results to the case of manifolds with nonnegative Ricci curvature and Euclidean volume growth. In the first part of this section we consider a *penalized* isoperimetric problem and we show that its associated isoperimetric profile converges locally uniformly to the isoperimetric profile of the manifold.

Throughout this section we are setting what follows. Let $(M^n,g)$ be a fixed complete Riemannian manifold, and let $o \in M$. Fix a sequence $\varepsilon _k\searrow 0$ and for any $k\in {\mathbb {N}}$ let $U_k$ be a bounded open smooth set in *M* containing the ball $B_{1/\varepsilon _k}(o)$ such that $U_k\subset U_{k+1}$; and choose $f_k\in C^\infty (M)$ such that

$$\begin{aligned} f_k\ge 0 , \qquad f_k|_{U_k} \equiv 0, \qquad |\nabla f_k|\le \varepsilon _k, \qquad f_{k+1}\le f_k, \end{aligned}$$

for any $k\in {\mathbb {N}}$. The previous choice can be made in such a way that for any *k* and for any $N>0$ there is $R>0$ such that $f_k>N$ on $M\setminus B_R(o)$. Define, for any $k\in {\mathbb {N}}$,

3.1 $$\begin{aligned} P_k(\Omega ) :=P(\Omega ) + \int _\Omega f_k \,\mathrm d{{\,\mathrm{vol}\,}}, \end{aligned}$$

for any set of finite perimeter $\Omega \subset M$, and

3.2 $$\begin{aligned} I_k(V) :=\inf \{ P_k(\Omega ) \ :\ \Omega \text {is a finite perimeter set with}\, {{\,\mathrm{vol}\,}}(\Omega ) = V\}, \end{aligned}$$

for any $V>0$. For simplicity, we shall denote by *I* the isoperimetric profile $I_{(M^n,g)}$.

### Lemma 3.1

Suppose that $(M^n,g)$ is noncollapsed and that $\mathrm {Ric}\ge (n-1)K$ for some $K \in {\mathbb {R}}$. Then the following two facts hold.

1. For any $k\in {\mathbb {N}}$ and any $V>0$ there exists a set of finite perimeter $\Omega$ of volume *V* such that $I_k(V) = P_k(\Omega )$.
2. For any $k\in {\mathbb {N}}$ the function $I_k:(0,+\infty )\rightarrow {\mathbb {R}}$ is continuous and the sequence $\{I_k\}_{k\in {\mathbb {N}}}$ converges from above to the isoperimetric profile $I_{(M^n,g)}$ locally uniformly on $(0,+\infty )$.

### Proof

For any *k* and $V>0$ the existence of a minimizer $\Omega$ for $P_k$ at volume *V* follows by means of the direct method. Indeed, a minimizing sequence $\{\Omega _i\}_{i\in {\mathbb {N}}}$ for $P_k$ at volume $V={{\,\mathrm{vol}\,}}(\Omega _i)$ has uniformly bounded perimeters, and thus it converges up to subsequence to some set $\Omega$ in $L^1_\mathrm{loc}(M)$ (see e.g. [59, Corollary 12.27], that clearly holds true also in a nonflat setting). The functional $P_k$ is lower semicontinuous with respect to $L^1_{\mathrm {loc}}$-convergence and if by contradiction ${{\,\mathrm{vol}\,}}(\Omega )<V$, since $f_k$ diverges at infinity, we would get that $\int _{\Omega _i}f_k \rightarrow +\infty$ as $i\rightarrow +\infty$.

For any *k* and $V>0$ denote by $\Omega ^k_V$ a minimizer of $P_k$ at volume *V*. First observe that $I_k$ is locally bounded; indeed for any $V>0$ the value of $I_k$ at volumes *v* in a neighborhood of *V* is estimated from above by the value of $P_k$ on balls of center *o* and given volume *v*.

Let us now prove that $I_k$ is continuous. Indeed, fix $V>0$ and let $V_i\rightarrow V$ be any sequence. Since $I_k(V_i) = P_k(\Omega _{V_i}^k)$, and thus the perimeters of $\Omega _{V_i}^k$ are uniformly bounded in *i*, we get that, as above, up to a subsequence, $\{\Omega _{V_i}^k\}_{i\in {\mathbb {N}}}$ converges in $L^1_\mathrm{loc}$ to some set $\Omega$. As $\{P_k(\Omega _{V_i}^k)\}_{i\in {\mathbb {N}}}$ is uniformly bounded, we deduce that ${{\,\mathrm{vol}\,}}(\Omega )=V$ and then the convergence $\Omega _{V_i}^k\rightarrow _i \Omega$ holds in $L^1$ on *M*. Moreover

$$\begin{aligned} I_k(V) \le P_k(\Omega ) \le \liminf _i P_k(\Omega _{V_i}^k) = \liminf _i I_k(V_i). \end{aligned}$$

Hence $I_k$ is lower semicontinuous. Let us now finish the proof of the continuity. Indeed, suppose by contradiction that $I_k(V) \le \limsup _i I_k(V_i) - \delta$ for some $\delta >0$. There exists a set *E* of volume *V* with $P_k(E)\le I_k(V) + \delta /4$, and then for large *i* we can find a ball $B_{r_i}(x_i)$ such that $r_i\rightarrow 0^+$ and either $E \cup B_{r_i}(x_i)$ or $E \setminus B_{r_i}(x_i)$ has volume $V_i$, and, denoting by $E_i$ such a set of volume $V_i$, we have

3.3 $$\begin{aligned} P_k(E_i) \le I_k(V) + \delta /2, \end{aligned}$$

for large *i*. This inequality follows from $P(E\cup B_{r_i}(x_i))\le P(E)+s(n,K,r_i)$, that is a consequence of Bishop-Gromov comparison, together with the fact that $r_i\rightarrow 0$. Finally, $P_k(E_i) \ge I_k(V_i)$, thus inserting the absurd hypothesis in (3.3) and passing to the limsup in *i* we derive a contradiction.

Therefore $\lim _i I_k(V_i) = I_k(V)$, and the arbitrariness of the sequence implies that $I_k$ is continuous at *V*, and then $I_k$ is continuous on $(0,+\infty )$.

Since $(M^n,g)$ is noncollapsed with Ricci bounded below, the isoperimetric profile *I* is continuous and strictly positive by [67, Corollary 2] and [13, Remark 4.7]. Since $I_k$ is continuous and $I\le I_{k+1}\le I_k$ for any $k\in {\mathbb {N}}$, if $I_k\rightarrow _k I$ pointwise, then the same convergence holds locally uniformly by Dini’s Monotone Convergence Theorem. Now for fixed $V>0$ we show, indeed, that $I_k(V)\rightarrow _k I(V)$. If by contradiction $\limsup _k I_k(V) \ge \delta + I(V)$ for some $\delta >0$, then one can consider a bounded smooth set $\Omega$ of volume *V* such that $P(\Omega ) \le I(V) + \delta /2$. Since $\Omega \Subset U_k$ for large *k*, we get that

$$\begin{aligned} I(V) + \frac{\delta }{2} \ge P(\Omega ) = \limsup _k P_k(\Omega ) \ge \delta + I(V), \end{aligned}$$

that is impossible. $\square$

### First and second variations of the penalized perimeter

In this section we derive first and second variations for the potential term in $P_k$, and we collect basic properties of the sets that minimize the penalized perimeter $P_k$ under a volume constraint. We recall that, if $E\subset M$ is a set of finite perimeter, then $D\chi _E= \nu _E {\mathcal {H}}^{n-1}\llcorner \partial ^*E$. The vector field $\nu _E$ is defined $|D\chi _E|$-a.e. and it is called *generalized interior unit normal*. In case $\Sigma \subset \partial ^*E$ is a smooth hypersurface, then $\nu _E$ coincides with the classical interior (with respect to *E*) unit normal along $\Sigma$.

Let us further recall that, if $E\subset M$ is a set of finite perimeter and *X* is a smooth compactly supported vector field on *M*, then we can define the *tangential divergence* $\mathrm{div}_E X :=\mathrm{div}X - \left\langle \nabla X (\nu _E), \nu _E \right\rangle$ at ${\mathcal {H}}^{n-1}$-a.e. point on $\partial ^*\Omega$. We say that *E* has *generalized (or distributional) mean curvature of E* *in direction* $\nu _E$ if there is $H_E \in L^1_\mathrm{loc}(|D\chi _E|)$ such that

$$\begin{aligned} \int _{\partial ^* E} \mathrm{div}_E X \,\mathrm d{\mathcal {H}}^{n-1} = - \int _{\partial ^* E} H_E\left\langle X,\nu _E\right\rangle \,\mathrm d{\mathcal {H}}^{n-1}, \end{aligned}$$

for every compactly supported vector field *X* on *M*. In such a case, the vector $H_E\nu _E$ is called *generalized (or, distributional) mean curvature of* *E*. Obviously, if $\Sigma \subset \partial ^* E$ is a smooth hypersurface, then tangential divergence and generalized mean curvature recover the corresponding classical quantities along $\Sigma$.

#### Lemma 3.2

Suppose that $(M^n,g)$ is noncollapsed and that $\mathrm {Ric}\ge (n-1)K$ for $K \in {\mathbb {R}}$. Let $\Omega$ be a minimizer of $P_k$, see (3.1), at volume *V*. Then $\Omega$ is bounded and there exists a constant $\uplambda =\uplambda _{k,V}\in {\mathbb {R}}$ such that

3.4 $$\begin{aligned} \frac{\mathrm d}{\mathrm dt} P_k(\phi _t(\Omega ))\bigg |_{t=0} = \uplambda \int _{\partial ^*\Omega } \left\langle X, - \nu _\Omega \right\rangle \,\mathrm d{\mathcal {H}}^{n-1}, \end{aligned}$$

for any smooth compactly supported vector field *X* on *M*, where $\phi _t$ is the flow of *X*.

Moreover, $\Omega$ has generalized mean curvature $H_\Omega$ in direction $\nu _\Omega$ given by

3.5 $$\begin{aligned} H_\Omega = \uplambda - f_k \qquad {\mathcal {H}}^{n-1} \text {-a.e. on} \partial ^*\Omega . \end{aligned}$$

Furthermore, there is an open representative of $\Omega$ and its topological boundary $\partial \Omega$ is given by the disjoint union $\partial \Omega = \partial _r\Omega \sqcup \partial _s\Omega$, where $\partial _r\Omega$ is a smooth hypersurface with topological boundary $\partial _s\Omega$ that is empty if the dimension is $n \le 7$ and with Hausdorff dimension less than or equal to $n-8$ when $n \ge 8$.

#### Proof

The first variation $\partial _t P_k(\phi _t(\Omega ))|_{t=0}$ vanishes whenever *X* generates a volume preserving variation, i.e., whenever $\int _{\partial ^*\Omega } \left\langle X,\nu _\Omega \right\rangle =0$. Arguing for example as in [59, Theorem 17.20] it is a classical matter to derive the existence of the Lagrange multiplier $\uplambda$ satisfying (3.4).

Being $\Omega , X, \phi _t$ as in the statement, we have that

$$\begin{aligned} \frac{\mathrm d}{\mathrm dt} \left( \int _{\Omega _t} f_k \,\mathrm d{{\,\mathrm{vol}\,}}\right) \bigg |_{t=0} = \int _{\partial ^* \Omega } f_k \left\langle X , - \nu _{\Omega } \right\rangle \,\mathrm d{\mathcal {H}}^{n-1}, \end{aligned}$$

where $\Omega _t:=\phi _t(\Omega )$. Such variation formula follows by an application of the area formula as in [59, Proposition 17.8], which can be readily reformulated on Riemannian manifolds. The first variation formula for the perimeter then yields that

$$\begin{aligned} \frac{\mathrm d}{\mathrm dt} P_k(\phi _t(\Omega ))\bigg |_{t=0} = \int _{\partial ^*\Omega } \left( \mathrm{div}X - \left\langle \nabla X (\nu _\Omega ), \nu _\Omega \right\rangle - f_k \left\langle X,\nu _\Omega \right\rangle \right) \,\mathrm d{\mathcal {H}}^{n-1}. \end{aligned}$$

We deduce that

$$\begin{aligned} \int _{\partial ^*\Omega } \mathrm{div}_{\Omega } X \,\mathrm d{\mathcal {H}}^{n-1} = \int _{\partial ^*\Omega } (f_k - \uplambda )\left\langle X,\nu _\Omega \right\rangle \,\mathrm d{\mathcal {H}}^{n-1}, \end{aligned}$$

which implies that $\Omega$ has generalized mean curvature $H_\Omega \nu _\Omega = - (f_k-\uplambda ) \nu _\Omega$.

The regularity of $\partial \Omega$ in the statement is classical, and it can be rigorously derived from the recent [14] (see also [50, 64, 83] also for the Euclidean case). Indeed, its consequences [14, Corollary 1.6 & Remark 1.7]. It implies that $\Omega$ has an open representative with topological boundary $\partial \Omega$ given by the disjoint union $\partial \Omega = \partial _r\Omega \sqcup \partial _s\Omega$, where $\partial _r\Omega$ is locally $C^{1,\alpha }$ and it has topological boundary $\partial _s\Omega$ with Hausdorff dimension less than or equal to $n-8$. Since $f_k$ is smooth, then (3.5) classically implies that the regular part $\partial _r\Omega$ is, in fact, smooth.

Now we can prove that $\Omega$ is bounded by a modification of a classical argument appeared in [76, Proposition 3.7] in the Euclidean setting and in [69, Theorem 3] on Riemannian manifolds (see also [13, Appendix B]). Fix $p_0 \in \partial _r \Omega$. Since $\partial _s\Omega$ is closed, there is $R_1>0$ such that $\partial \Omega \cap B_{R_1}(p_0)$ is a nonempty smooth hypersurface, and it is well defined the inner normal $\nu _\Omega$ of $\Omega$ on $\partial \Omega \cap B_{R_1}(p_0)$. Let $\varphi \in C^\infty _c(\partial \Omega \cap B_{R_1}(p_0))$ be a nonvanishing nonnegative function and consider the normal vector field $X=-\varphi \nu _\Omega$. Let $\Phi _t(x):=\exp (tX(x))$ and let $\Omega _t$ be the varied set whose essential boundary is $\Phi _t(\partial ^*\Omega )$, for $|t|<\tau$ and $\tau >0$. Since $\alpha :=\int _{\partial \Omega } \left\langle X,-\nu _\Omega \right\rangle >0$, first variation formulae for perimeter and volume give that

3.6 $$\begin{aligned} \begin{aligned} {{\,\mathrm{vol}\,}}(\Omega _t)&= {{\,\mathrm{vol}\,}}(\Omega ) + \alpha t + O(t^2),\\ P(\Omega _t,B_{R_1}(p_0))&= P(\Omega ,B_{R_1}(p_0)) + t \int _{\partial \Omega } \varphi H_\Omega \,\mathrm d{\mathcal {H}}^{n-1} + O(t^2), \end{aligned} \end{aligned}$$

for $|t|<\tau$. Moreover, as $\varphi >0$, we have that

3.7 $$\begin{aligned} \Omega \subset \Omega _t \qquad \text {for} 0\le t<\tau , \end{aligned}$$

up to decreasing $\tau$. As $\alpha >0$ and $|\int \varphi H_\Omega |<+\infty$, (3.6) and (3.7) imply that there exist $\varepsilon _0>0$ and $\beta >0$ such that for any $\varepsilon \in (-\varepsilon _0,\varepsilon _0)$ there is a finite perimeter set *E* with

3.8 $$\begin{aligned} \begin{aligned}&E\Delta \Omega \Subset B_{R_1}(p_0),\\&{{\,\mathrm{vol}\,}}(E)={{\,\mathrm{vol}\,}}(\Omega )+\varepsilon ,\\&P(E,B_{R_1}(p_0)) \le P(\Omega ,B_{R_1}(p_0))+ \beta |\varepsilon |, \\&\varepsilon >0 \text { implies } \Omega \subset E. \end{aligned} \end{aligned}$$

Now for $r>R_1$ let

$$\begin{aligned} V(r):={{\,\mathrm{vol}\,}}(\Omega \setminus B_r(p_0)), \qquad A(r):=P(\Omega , M\setminus B_r(p_0)). \end{aligned}$$

Since *M* is noncollapsed and the Ricci curvature is bounded from below, an isoperimetric inequality holds true for sufficiently small volumes [52, Lemma 3.2], i.e. there is $v_0>0$ such that

$$\begin{aligned} c_0{{\,\mathrm{vol}\,}}(\Omega )^{(n-1)/n}\le P(\Omega ), \end{aligned}$$

holds true with some $c_0 > 0$ for any finite perimeter set $\Omega \subset M^n$ with ${{\,\mathrm{vol}\,}}(\Omega )<v_0$. Hence for some $R_2>R_1$, for $r\ge R_2>0$ we can apply such an isoperimetric inequality on $\Omega \setminus B_r(p_0)$, so that

$$\begin{aligned}&| V'(r) | + A(r) = {\mathcal {H}}^{n-1}(\partial B_r(p_0) \cap \Omega ) + P(\Omega , M\setminus B_r(p_0))\\&= P(\Omega \setminus B_r(p_0)) \ge c_0 V(r)^{\frac{n-1}{n}}, \end{aligned}$$

for some $c_0>0$ for almost every $r\ge R_2$. We want to prove that

3.9 $$\begin{aligned} A(r) \le | V'(r) | + C V(r) , \end{aligned}$$

for some constant *C*, and for almost every *r* sufficiently big. Combining with the previous inequality, in this way we would get

$$\begin{aligned} c_0 V(r)^{\frac{n-1}{n}} \le C V(r) + 2| V'(r) | \le \frac{c_0}{2}V(r)^{\frac{n-1}{n}} - 2 V'(r), \end{aligned}$$

because $$| V'(r) | = - V'(r) $$ and $$ C V(r)\le \tfrac{c_0}{2}V(r)^{\frac{n-1}{n}} $$ for almost every sufficiently big radius. Hence ODE comparison implies that *V*(*r*) vanishes at some $$r={\overline{r}}<+\infty $$, i.e., $$\Omega $$ is bounded as a set of finite perimeter.

So we are left to prove (3.9). For some $R_3\ge R_2$, for $r\ge R_3$ we can assume that $V(r)<\varepsilon _0$. For fixed $r\ge R_3$ let $\varepsilon :=V(r)$ and assume also that ${\mathcal {H}}^{n-1}(\partial \Omega \cap \partial B_r(p_0))=0$ and $V'(r)$ exists, which hold for almost every *r*. Given such $\varepsilon$, there exists *E* as in (3.8). Finally let $F= E \cap B_r(p_0)$. It follows that ${{\,\mathrm{vol}\,}}(F) = {{\,\mathrm{vol}\,}}(\Omega )$ and (3.8) implies that

3.10 $$\begin{aligned} \Omega \cap B_r(p_0) \subset F, \qquad F\setminus \Omega \subset B_{R_1}(p_0), \qquad {{\,\mathrm{vol}\,}}(F\setminus \Omega ) = \varepsilon . \end{aligned}$$

Since $\Omega$ is a minimizer for $P_k$ at volume ${{\,\mathrm{vol}\,}}(\Omega )={{\,\mathrm{vol}\,}}(F)$, we deduce

3.11 $$\begin{aligned} \begin{aligned} P_k(\Omega )&\le P_k(F) = P(E) - P(\Omega ,M\setminus B_r(p_0)) + {\mathcal {H}}^{n-1}(\partial B_r(p_0) \cap \Omega ) + \int _F f_k\,\mathrm d{{\,\mathrm{vol}\,}}\\&\le P(\Omega ) + \beta \varepsilon - A(r) + |V'(r)| + \int _F f_k\,\mathrm d{{\,\mathrm{vol}\,}}. \end{aligned} \end{aligned}$$

Up to increase $R_3$, we can assume that $\inf _{M\setminus B_r(p_0)} f_k > \sup _{B_{R_1}(p_0)} f_k$. By (3.10) we then get

$$\begin{aligned} \begin{aligned} \int _F f_k\,\mathrm d{{\,\mathrm{vol}\,}}&= \int _{F\cap \Omega } f_k\,\mathrm d{{\,\mathrm{vol}\,}}+ \int _{F\setminus \Omega } f_k\,\mathrm d{{\,\mathrm{vol}\,}}\le \int _{\Omega \cap B_r(p_0)} f_k\,\mathrm d{{\,\mathrm{vol}\,}}+ \varepsilon \sup _{B_{R_1}(p_0)} f_k \\&\le \int _{\Omega \cap B_r(p_0)} f_k\,\mathrm d{{\,\mathrm{vol}\,}}+\int _{\Omega \setminus B_r(p_0)} f_k\,\mathrm d{{\,\mathrm{vol}\,}}= \int _\Omega f_k \,\mathrm d{{\,\mathrm{vol}\,}}\end{aligned} \end{aligned}$$

Combining the latter one with (3.11) we obtain (3.9) with $C=\beta$. $\square$

We also need the following second variation formula for the potential term.

#### Lemma 3.3

Let *M* be a complete Riemannian manifold. Let $\Omega \subset M$ be a set of finite perimeter and suppose that there is a smooth hypersurface $\Sigma$ with boundary contained in the essential boundary $\partial ^*\Omega$. Let $X:\Sigma \rightarrow TM$ be a smooth vector field compactly supported in the interior of $\Sigma$ and denote $\phi _t(x):=\exp (tX(x))$ for $x \in \Sigma$.

For some $\tau >0$, if $\Omega _t$ is the set of finite perimeter with essential boundary $\phi _t(\partial ^*\Omega )$ for $|t|<\tau$ and $X_t:=\partial _t \phi _t$ is the variation field along $\phi _t(\Sigma )$, then

3.12 $$\begin{aligned} \frac{\mathrm d}{\mathrm dt} \left( \int _{\Omega _t} f_k \,\mathrm d{{\,\mathrm{vol}\,}}\right) = \int _{\partial ^* \Omega _t} f_k \left\langle X_t , - \nu _{\Omega _t} \right\rangle \,\mathrm d{\mathcal {H}}^{n-1} \qquad \forall \,|t|<\tau , \end{aligned}$$

If also *X* is normal along $\Sigma$, then

3.13 $$\begin{aligned} \begin{aligned} \frac{\mathrm d^2}{\mathrm dt^2} \left( \int _{\Omega _t} f_k \,\mathrm d{{\,\mathrm{vol}\,}}\right) \bigg |_{t=0} =&\int _\Sigma \left( f_k H_\Sigma \left\langle X,\nu _\Omega \right\rangle ^2 - \left\langle \nabla f_k , X\right\rangle \left\langle X, \nu _\Omega \right\rangle \right) \,\mathrm d{\mathcal {H}}^{n-1}, \end{aligned} \end{aligned}$$

where $H_\Sigma$ is the mean curvature of $\Sigma$ in direction $\nu _\Omega$.

#### Proof

The first variation (3.12) follows by an application of the area formula as in [59, Proposition 17.8], which can be readily adapted on Riemannian manifolds. The second variation (3.13) follows by differentiating (3.12). Indeed, rewriting the varied boundary $\partial ^*\Omega _t$ as an embedding $F_t:\Sigma \rightarrow M$, where $F_t(\Sigma )= \phi _t(\Sigma )$, we have

$$\begin{aligned} \frac{\mathrm d}{\mathrm dt} \left( \int _{\Omega _t} f_k \,\mathrm d{{\,\mathrm{vol}\,}}\right) = \int _{\Sigma } [f_k \left\langle X_t , - \nu _{\Omega _t} \right\rangle ]\circ F_t \,\mathrm d{{\,\mathrm{vol}\,}}(t), \end{aligned}$$

where ${{\,\mathrm{vol}\,}}(t)$ is the volume form on $\Sigma$ induced by $F_t$. Hence

$$\begin{aligned} \begin{aligned} \frac{\mathrm d^2}{\mathrm dt^2} \left( \int _{\Omega _t} f_k \,\mathrm d{{\,\mathrm{vol}\,}}\right) =&\int _\Sigma - \left\langle \nabla f_k , X_t\right\rangle \left\langle X_t, \nu _{\Omega _t}\right\rangle \circ F_t - f_k \left\langle \nabla _{X_t} X_t, \nu _{\Omega _t}\right\rangle \circ F_t +\\&- f_k \left\langle X_t, \nabla _{X_t} \nu _{\Omega _t}\right\rangle \,\mathrm d{{\,\mathrm{vol}\,}}(t) + \int _\Sigma f_k\left\langle X_t,-\nu _{\Omega _t}\right\rangle \circ F_t \, \partial _t \,\mathrm d{{\,\mathrm{vol}\,}}(t) . \end{aligned} \end{aligned}$$

Since $\nabla _{X_t}X_t=0$ and $\nabla _{X_t}\nu _{\Omega _t}$ is tangent along $F_t(\Sigma )$, evaluating at $t=0$ we get

3.14 $$\begin{aligned}&\frac{\mathrm d^2}{\mathrm dt^2} \left( \int _{\Omega _t} f_k \,\mathrm d{{\,\mathrm{vol}\,}}\right) \bigg |_{t=0}= \int _\Sigma - \left\langle \nabla f_k , X\right\rangle \left\langle X, \nu _{\Omega }\right\rangle \,\mathrm d{\mathcal {H}}^{n-1}\nonumber \\&+ \int _\Sigma f_k\left\langle X,-\nu _{\Omega }\right\rangle \, \partial _t \,\mathrm d{{\,\mathrm{vol}\,}}(t)|_{t=0} . \end{aligned}$$

Since *X* is normal along $\Sigma$, i.e., its tangent projection $X^\top$ onto $T\Sigma$ vanishes, the identity (3.13) follows by the fact that

3.15 $$\begin{aligned} \partial _t \,\mathrm d{{\,\mathrm{vol}\,}}(t)|_{t=0} = \left( \mathrm{div}_{\Sigma } ( X^\top ) - H_\Sigma \left\langle X, \nu _\Omega \right\rangle \right) \,\mathrm d{\mathcal {H}}^{n-1}\llcorner \Sigma = - H_\Sigma \left\langle X, \nu _\Omega \right\rangle \,\mathrm d{\mathcal {H}}^{n-1}\llcorner \Sigma . \end{aligned}$$

$$\square $$

In the hypotheses and notation of Lemma 3.3, if also $\Omega$ has generalized mean curvature $H_\Omega \nu _\Omega$, if $\varphi$ is a smooth function compactly supported in $\Sigma \subset \partial ^*\Omega$, we can plug in the normal field $X= -\varphi \nu _\Omega$, so that (3.13) reads

3.16 $$\begin{aligned} \frac{\mathrm d^2}{\mathrm dt^2} \left( \int _{\Omega _t} f_k \,\mathrm d{{\,\mathrm{vol}\,}}\right) \bigg |_{t=0} = \int _{\partial ^*\Omega } \left( \varphi ^2 f_k H_\Omega - \varphi ^2 \left\langle \nabla f_k , \nu _\Omega \right\rangle \right) \,\mathrm d{\mathcal {H}}^{n-1}, \end{aligned}$$

indeed $$H_\Sigma =H_\Omega $$ on $$\Sigma $$.

### Proof of Theorem 1.4

We will need the following well known lemma that, roughly speaking, gives an approximation in $H^1$ of the function identically equal to 1 on the boundary of a set, provided its singular part is sufficiently small. We will apply such a result to a volume constrained minimizer of $P_k$.

#### Lemma 3.4

Let $\Omega \subset M^n$ be a bounded open set such that its topological boundary $\partial \Omega$ is given by the disjoint union $\partial \Omega = \partial _r\Omega \sqcup \partial _s\Omega$, where $\partial _r\Omega$ is a smooth hypersurface with topological boundary $\partial _s\Omega$ and the Hausdorff dimension of $\partial _s\Omega$ is less than or equal to $n-3$.

Then for any $\delta >0$ there is $\varphi _\delta \in C^\infty _c(\partial _r \Omega )$ such that

$$\begin{aligned} 0\le \varphi _\delta \le 1, \qquad \lim _{\delta \rightarrow 0} \int _{\partial \Omega } \varphi _\delta ^2\,\mathrm d{\mathcal {H}}^{n-1} = P(\Omega ), \qquad \lim _{\delta \rightarrow 0}\int _{\partial \Omega } |\nabla \varphi _\delta |^2\,\mathrm d{\mathcal {H}}^{n-1} =0, \end{aligned}$$

where $\nabla \varphi _\delta$ here denotes the gradient of $\varphi _\delta$ as a function on the submanifold $\partial _r\Omega$.

The above lemma has been proved in the Euclidean setting in [78, Lemma 2.4] under additional regularity assumptions first, and then extended to Riemannian manifolds in [20, Proposition 2.5, Remark 2.6]. It is observed in [66, Lemma 3.1] that the regularity assumptions on $\partial \Omega$ as in the statement are sufficient (see also [21, Lemma 3.1]).

In the following proof of Theorem 1.4, we are making variations of a volume constrained minimizer $\Omega$ for the penalized perimeter along the normal field $X = - \varphi \nu _\Omega$, with $\varphi$ given by Lemma 3.4. We point out that in dimension $n \le 7$, by means of the regularity result recalled in Lemma 3.2, one could safely consider $X = - \nu _\Omega$.

#### Conclusion of the proof of Theorem 1.4

For the ease of notation we do not explicitly write the volume forms under the integral sign, since it will be clear from the context.

Let $\Omega =\Omega (k,{\widetilde{V}})$ be a fixed minimizer of volume ${\widetilde{V}}$ for $P_k$. Let $\varphi \in C^\infty _c(\partial _r\Omega )$ be a smooth nonvanishing function with $0\le \varphi \le 1$ supported in the regular part of $\partial \Omega$ and define the normal field $X=-\varphi \nu _\Omega$ and $\phi _t(x):=\exp (tX(x))$ for $x \in \partial ^*\Omega$. Denote by $\Omega _t$ the varied set whose reduced boundary is $\phi _t(\partial ^*\Omega )$, for $|t|<\tau$ and $\tau >0$. Define

$$\begin{aligned} v(t):={{\,\mathrm{vol}\,}}(\Omega _t), \qquad J(v):=P_k(\Omega _t) \quad \text {for the unique} t \text {s.t.} v=v(t). \end{aligned}$$

The definitions of *v*(*t*) and *J*(*v*) are well posed, indeed

$$\begin{aligned} v'(t) = \int _{\partial ^*\Omega _t} \left\langle -\nu _{\Omega _t}, \partial _t \phi _t\right\rangle , \end{aligned}$$

and then $v'(0)=\int _{\partial \Omega }\varphi >0$ and $v'>0$ for every *t* if $\tau$ is small enough. Hence *v* is locally invertible and *J*(*v*) is well defined for $v \in ({\widetilde{V}} - {\widetilde{\delta }}, {\widetilde{V}} + {\widetilde{\delta }})$ for some ${\widetilde{\delta }} >0$. Let $v^{-1}$ denote the inverse of *v*(*t*). We thus have

3.17 $$\begin{aligned} \begin{aligned} \frac{\mathrm d}{\mathrm dt}P(\Omega _t)\bigg |_{t=0}&= \int _{\partial \Omega } \varphi H_\Omega ,\\ (v^{-1})'(v)&= \left( \int _{\partial ^*\Omega _t} \left\langle -\nu _{\Omega _t}, \partial _t \phi _t\right\rangle \right) ^{-1},\\ (v^{-1})'({\widetilde{V}})&= \left( \int _{\partial \Omega } \varphi \right) ^{-1},\\ (v^{-1})''({\widetilde{V}})&= -\frac{1}{\left( \int _{\partial \Omega } \varphi \right) ^2}(v^{-1})'({\widetilde{V}}) v''(0) =-\frac{1}{\left( \int _{\partial \Omega } \varphi \right) ^3}\int _{\partial \Omega } \varphi ^2 H_\Omega , \end{aligned} \end{aligned}$$

where the last equality follows by computing $v''(0)$ analogously as in (3.14) and (3.15). Observing that $J(v)=P_k(\Omega _{v^{-1}(v)})$, we can compute

$$\begin{aligned} \begin{aligned} \left( \int _{\partial \Omega } \varphi \right) ^2&\frac{\mathrm d^2}{\mathrm dv^2} J(v) \bigg |_{v={\widetilde{V}}} \\ {}&= \left( \int _{\partial \Omega } \varphi \right) ^2\frac{\mathrm d}{\mathrm dv} \left( (v^{-1})' \left( \frac{\mathrm d}{\mathrm dt}P_k(\Omega _t) \right) \circ v^{-1} \right) \bigg |_{v={\widetilde{V}}} \\&=\left( \int _{\partial \Omega } \varphi \right) ^2 \left( (v^{-1})''({\widetilde{V}}) \frac{\mathrm d}{\mathrm dt}P_k(\Omega _t)\bigg |_{t=0} + [(v^{-1})'({\widetilde{V}})]^2 \frac{\mathrm d^2}{\mathrm dt^2}P_k(\Omega _t)\bigg |_{t=0}\right) . \end{aligned} \end{aligned}$$

Using (3.12), (3.16), and (3.17) we get

$$\begin{aligned} \begin{aligned} \left( \int _{\partial \Omega } \varphi \right) ^2\frac{\mathrm d^2}{\mathrm dv^2} J(v) \bigg |_{v={\widetilde{V}}} =&-\frac{1}{\int _{\partial \Omega } \varphi }\int _{\partial \Omega } \varphi ^2 H_\Omega \left( \int _{\partial \Omega } \varphi H_\Omega + \frac{\mathrm d}{\mathrm dt}\int _{\Omega _t} f_k \bigg |_{t=0} \right) + \\&+\frac{\mathrm d^2}{\mathrm dt^2}P(\Omega _t)\bigg |_{t=0} + \frac{\mathrm d^2}{\mathrm dt^2}\int _{\Omega _t} f_k \bigg |_{t=0} \\ =&-\frac{1}{\int _{\partial \Omega } \varphi }\int _{\partial \Omega } \varphi ^2 H_\Omega \left( \int _{\partial \Omega } \varphi H_\Omega +\int _{\partial \Omega } \varphi f_k \right) +\\&+\int _{\partial \Omega } \varphi ^2 f_k H_\Omega - \varphi ^2 \left\langle \nabla f_k , \nu _\Omega \right\rangle + \frac{\mathrm d^2}{\mathrm dt^2}P(\Omega _t)\bigg |_{t=0}. \end{aligned} \end{aligned}$$

The second variation formula for the perimeter yields

$$\begin{aligned} \frac{\mathrm d^2}{\mathrm dt^2}P(\Omega _t)\bigg |_{t=0}= \int _{\partial \Omega } |\nabla \varphi |^2 + \varphi ^2 \left( H_\Omega ^2 - |A_\Omega |^2 - \mathrm {Ric}(\nu _\Omega ,\nu _\Omega )\right) , \end{aligned}$$

where $A_\Omega$ is the second fundamental form of $\partial _r \Omega$ and $\nabla \varphi$ is the gradient of $\varphi$ as a function on the submanifold $\partial _r\Omega$. Since $\mathrm {Ric}\ge (n-1)K$, $|\nabla f_k|\le \varepsilon _k$, $\varphi \le 1$, and $H_\Omega + f_k = \uplambda \equiv \uplambda _{k,{\widetilde{V}}} \in {\mathbb {R}}$ at ${\mathcal {H}}^{n-1}$-almost every point on $\partial ^*\Omega$, we estimate

$$\begin{aligned} \begin{aligned} \left( \int _{\partial \Omega } \varphi \right) ^2&\frac{\mathrm d^2}{\mathrm dv^2} J(v) \bigg |_{v={\widetilde{V}}}\\ \le&-\frac{1}{\int _{\partial \Omega } \varphi }\int _{\partial \Omega } \varphi ^2 H_\Omega \left( \int _{\partial \Omega }\varphi ( H_\Omega + f_k) \right) + \int _{\partial \Omega } \varphi ^2 f_k H_\Omega + \varepsilon _k P(\Omega ) +\\&+\int _{\partial \Omega }\varphi ^2 H_\Omega ^2 +(n-1)|K|P(\Omega ) - \int _{\partial \Omega } \varphi ^2|A_\Omega |^2 + \int _{\partial \Omega } |\nabla \varphi |^2\\ =&-\uplambda \int _{\partial \Omega } \varphi ^2H_\Omega + \int _{\partial \Omega } (f_k + H_\Omega )\varphi ^2H_\Omega + \varepsilon _k P(\Omega ) +\\&+(n-1)|K|P(\Omega )- \int _{\partial \Omega } \varphi ^2|A_\Omega |^2 + \int _{\partial \Omega } |\nabla \varphi |^2\\ =&(\varepsilon _k + (n-1)|K|)P(\Omega ) - \int _{\partial \Omega } \varphi ^2|A_\Omega |^2 + \int _{\partial \Omega } |\nabla \varphi |^2. \end{aligned} \end{aligned}$$

Hence

3.18 $$\begin{aligned} J''({\widetilde{V}}) \le (\varepsilon _k + (n-1)|K|)\frac{P(\Omega )}{\left( \int _{\partial \Omega } \varphi \right) ^2} +\frac{1}{\left( \int _{\partial \Omega } \varphi \right) ^2} \left( \int _{\partial \Omega } |\nabla \varphi |^2 - \int _{\partial \Omega } \varphi ^2 |A_\Omega |^2 \right) . \end{aligned}$$

By definition of $I_k$ we have that $I_k(v) \le J(v)$ for any $v \in ({\widetilde{V}} - {\widetilde{\delta }}, {\widetilde{V}} + {\widetilde{\delta }})$ and $I_k({\widetilde{V}}) = J({\widetilde{V}})$. Then by the latter observation,

$$\begin{aligned} \begin{aligned} {\overline{D}}^2 I_k ({\widetilde{V}}) :=&\limsup _{h\rightarrow 0} \frac{I_k({\widetilde{V}} +h) + I_k({\widetilde{V}} - h) - 2 I_k({\widetilde{V}})}{h^2} \le J''({\widetilde{V}}). \end{aligned} \end{aligned}$$

Now observe that we could take $\varphi =\varphi _\delta$ for any $\delta$ with $\varphi _\delta$ as in Lemma 3.4. Hence letting $\delta \rightarrow 0$, using that $P(\Omega ) \ge I({\widetilde{V}})$, and exploiting (3.18), we conclude that

3.19 $$\begin{aligned} {\overline{D}}^2 I_k ({\widetilde{V}}) \le \frac{\varepsilon _k + (n-1)|K|}{P(\Omega )} \le \frac{\varepsilon _k + (n-1)|K|}{I({\widetilde{V}})}. \end{aligned}$$

Recalling that *I* is continuous and strictly positive ( [67, Corollary 2] and [13, Remark 4.7]), up to taking a smaller ${\widetilde{\delta }}$, we have that *I*(*V*) is bounded below by a strictly positive constant independent of $V \in ({\widetilde{V}} - {\widetilde{\delta }}, {\widetilde{V}} + {\widetilde{\delta }})$. Since ${\widetilde{V}}$ above is arbitrary, we deduce that ${\overline{D}}^2 I_k (V) \le 2C_{{\widetilde{V}}}<+\infty$ for every $V \in ({\widetilde{V}} - {\widetilde{\delta }}, {\widetilde{V}} + {\widetilde{\delta }})$. The latter, together with the fact that $I_k$ is continuous, see Lemma 3.1, implies that $I_k-C_{{\widetilde{V}}}V^2$ is concave on $({\widetilde{V}} - {\widetilde{\delta }}, {\widetilde{V}} + {\widetilde{\delta }})$.

Passing to the limit as $k\rightarrow +\infty$, as $I_k\rightarrow _k I$ locally uniformly by Lemma 3.1, we deduce that $I-C_{{\widetilde{V}}}V^2$ is concave on $({\widetilde{V}} - {\widetilde{\delta }}, {\widetilde{V}} + {\widetilde{\delta }})$ as well. Moreover passing to the limit as $k\rightarrow +\infty$ in (3.19), taking into account [19, Lemma B.3.11], we conclude (1.3). Finally, the inequality for the second incremental ratios (1.3) is known to be equivalent to the distributional inequality (1.4), see [19]. Moreover since the function *I* is locally $C_{{\widetilde{V}}}$-concave around any ${\widetilde{V}}\in (0,\mathrm {vol}(M^n))$, it is twice differentiable almost everywhere and hence from (1.3) the inequality

$$\begin{aligned} I''({\widetilde{V}}) \le \frac{(n-1)|K|}{I({\widetilde{V}})}, \end{aligned}$$

follows for almost all $V\in (0,\mathrm {vol}(M^n))$, thus proving (1.5). $\square$

It would be interesting to understand how the above proof could be improved in order to get concavity properties on the function $I_{(M^n,g)}^{\frac{n}{n-1}}$. Under the a priori assumption of existence of isoperimetric sets, it has been studied in [18–21, 63].

### Consequences of the concavity properties of the isoperimetric profile

In this section we collect some consequences of Theorem 1.4. First, since concave functions are locally Lipschitz, we deduce the following useful corollary, which improves the main result in [67, Theorem 2].

#### Corollary 3.5

Suppose that $(M^n,g)$ is noncollapsed and that $\mathrm {Ric}\ge (n-1)K$ for some $K \in {\mathbb {R}}$. Then the isoperimetric profile $I_{(M^n,g)}$ is a locally Lipschitz function away from 0.

In the following result we exploit the concavity of *I* under nonnegative Ricci curvature, together with the sharp isoperimetric inequality (2.8), to deduce the precise asymptotic expansion of *I* and $I'$ at infinity when $\mathrm {Ric}\ge 0$.

#### Corollary 3.6

Let $(M^n,g)$ be a noncompact Riemannian manifold with $\mathrm {Ric}\ge 0$, and denote by *I* its isoperimetric profile. Then

3.20 $$\begin{aligned} \lim _{v\rightarrow +\infty }\frac{I(v)}{v^{(n-1)/n}}=n(\omega _n\mathrm {AVR}(M^n,g))^{1/n}. \end{aligned}$$

Moreover the right derivative $I'_+(v):=\lim _{h\rightarrow 0^+} \frac{I(v+h)-I(v)}{h}$ exists at any $v>0$ and it satisfies

3.21 $$\begin{aligned} \lim _{v\rightarrow +\infty }v^{1/n}I'_+(v)=(n-1)(\omega _n\mathrm {AVR}(M^n,g))^{1/n}. \end{aligned}$$

#### Proof

For simplicity, throughout the proof we denote $\theta :=\mathrm {AVR}(M^n,g)$. Let us first prove (3.20). We first deal with the case $\theta >0$. Let us fix $p\in M^n$. From Bishop–Gromov comparison theorem, see Theorem 2.11, we know that $P(B_r(p))/(n\omega _nr^{n-1})$ is almost everywhere non-increasing on $[0,+\infty )$. Since ${{\,\mathrm{vol}\,}}(B_r(p))/(\omega _nr^n)\rightarrow \theta$, as $r\rightarrow +\infty$, a standard use of the coarea formula for ${{\,\mathrm{vol}\,}}(B_r(p))$, together with the monotonicity ensured by the Bishop–Gromov comparison result, imply that the essential limit of $P(B_r(p))/(n\omega _nr^{n-1})$ as $r\rightarrow +\infty$ is $\theta$ as well. Hence, for every $\varepsilon >0$ there exists $r_0:=r_0(\varepsilon )$ such that for almost every $r\ge r_0$ we have

$$\begin{aligned} P(B_r(p))\le (1+\varepsilon )\theta n\omega _n r^{n-1}. \end{aligned}$$

Moreover, by approximating balls from the outside and by using the semicontinuity of the perimeter, the previous inequality holds for *all* $r\ge r_0$. Hence for every *V* sufficiently large (recall that our manifold has infinite volume), there exists a ball of center *p*, radius $r:=r(V)\ge r_0$ and volume *V* such that

$$\begin{aligned} I(V)\le P(B_r(p))\le (1+\varepsilon )\theta n\omega _nr^{n-1}\le (1+\varepsilon )n(\omega _n\theta )^{1/n}V^{(n-1)/n}, \end{aligned}$$

where in the last inequality we used $\theta \omega _nr^n\le V$, which comes from Bishop–Gromov comparison. Since $I(V)\ge n(\omega _n\theta )^{1/n}V^{(n-1)/n}$ for every $V>0$, as an immediate outcome of Theorem 2.21, we get (3.20) in the case $\theta >0$.

Let us now prove (3.20) when $\theta =0$. For every $V>0$ there exists a radius $r:=r(V)$ such that ${{\,\mathrm{vol}\,}}(B_{r(V)}(p))=V$. Notice that $r(V)\rightarrow +\infty$ when $V\rightarrow +\infty$. Moreover

3.22 $$\begin{aligned} \begin{aligned} \frac{I(V)}{V^{(n-1)/n}}&\le \frac{P(B_{r}(p))}{{{\,\mathrm{vol}\,}}(B_{r}(p))^{(n-1)/n}}=\frac{P(B_r(p))}{{{\,\mathrm{vol}\,}}(B_r(p))}\left( \frac{{{\,\mathrm{vol}\,}}(B_r(p))}{\omega _n r^n}\right) ^{1/n}r\omega _n^{1/n}\\&\le n\omega _n^{1/n}\left( \frac{{{\,\mathrm{vol}\,}}(B_r(p))}{\omega _n r^n}\right) ^{1/n}, \end{aligned} \end{aligned}$$

where in the first inequality we used the definition of isoperimetric profile, and in the last inequality we used the comparison in (2.3). Since $\theta =0$ we have that for every $\varepsilon$ there exists $r_0:=r_0(\varepsilon )$ such that ${{\,\mathrm{vol}\,}}(B_r(p))/(\omega _nr^n)\le \varepsilon$ for every $r\ge r_0$. Hence from (3.22) we conclude the sought limit in (3.20) also in the case $\theta =0$.

Now we can prove (3.21). If *M* is not noncollapsed, then (3.21) is trivially verified due to the equivalence in Proposition 2.18. So let us prove (3.21) when $M^n$ is noncollapsed. By Theorem 1.4 we know that *I* is concave, which implies the existence of $I'_+$ everywhere and that $I'_+$ is nonincreasing.

We claim that, for any $\varepsilon >0$ and $0<\delta <2$ there exists ${\bar{v}}= {\bar{v}}(\varepsilon , \delta )$ such that

3.23 $$\begin{aligned} \left| \frac{I(v(1+\delta )) - I(v)}{(v(1+\delta ))^{\frac{n-1}{n}} - v^{\frac{n-1}{n}} } - n(\omega _n \theta )^{1/n}\right| \le \varepsilon \quad \text {for any}\, v\ge {\bar{v}}. \end{aligned}$$

Indeed the latter follows from (3.20) and the inequality

$$\begin{aligned}&\left| \frac{I(v(1+\delta )) - I(v)}{(v(1+\delta ))^{\frac{n-1}{n}} - v^{\frac{n-1}{n}} } - n(\omega _n \theta )^{1/n}\right| \\ {}&\le \frac{(1+\delta )^{\frac{n-1}{n}}}{(1+\delta )^{\frac{n-1}{n}}-1}\left| \frac{I(v(1+\delta ))}{(v(1+\delta ))^{\frac{n-1}{n}}} - n(\omega _n \theta )^{1/n}\right| + \frac{1}{(1+\delta )^{\frac{n-1}{n}}-1}\left| \frac{I(v)}{v^{\frac{n-1}{n}}} - n(\omega _n \theta )^{1/n}\right| \, . \end{aligned}$$

The monotonicity of $I_+'$ gives

3.24 $$\begin{aligned} I(v(1+\delta )) - I(v) = \int _{v}^{v(1+\delta )} I'_+(t) dt \ge \delta v I_+'(v(1+\delta )) \, , \end{aligned}$$

and

3.25 $$\begin{aligned} I(v(1+\delta )) - I(v) = \int _{v}^{v(1+\delta )} I'_+(t) dt \le \delta v I_+'(v) \, , \end{aligned}$$

for all $v>0$, where we also employed the fact that *I* is Lipschitz, as observed in Corollary 3.5, in order to apply the Fundamental Theorem of Calculus. Hence, we get

3.26 $$\begin{aligned} \begin{aligned} \frac{I(v(1+\delta )) - I(v)}{(v(1+\delta ))^{\frac{n-1}{n}} - v^{\frac{n}{n-1}}}&\ge \frac{\delta v\, I'_+(v(1+\delta ))}{\frac{n-1}{n}\int _v^{v(1+\delta )} t^{-1/n} dt}\\&\ge (1+\delta )^{-1/n}\frac{n}{n-1} (v(1+\delta ))^{1/n} I'_+(v(1+\delta )) \, , \end{aligned} \end{aligned}$$

and

3.27 $$\begin{aligned} \frac{I(v(1+\delta )) - I(v)}{(v(1+\delta ))^{\frac{n-1}{n}} - v^{\frac{n}{n-1}}} \le \frac{\delta v\, I'_+(v)}{\frac{n-1}{n}\int _v^{v(1+\delta )} t^{-1/n} dt} \le (1+\delta )^{1/n} \frac{n}{n-1} v^{1/n} I'_+(v) \, , \end{aligned}$$

for all $v>0$. The latter, together with (3.23), implies

$$\begin{aligned} (1+\delta )^{-1/n} (n-1)&(\omega _n\theta )^{1/n} - C(n)\varepsilon \\ {}&\le \liminf _{v\rightarrow \infty } v^{1/n}I_+'(v) \\ {}&\le \limsup _{v\rightarrow \infty } v^{1/n}I_+'(v) \\ {}&\le (1+\delta )^{1/n} (n-1)(\omega _n\theta )^{1/n} + C(n)\varepsilon \, . \end{aligned}$$

Letting $\varepsilon , \delta \rightarrow 0$ we get the sought conclusion. $\square$

#### Remark 3.7

We remark that (3.20) holds for arbitrary ${{\mathsf {C}}}{{\mathsf {D}}}(0,n)$ spaces of infinite volume since it only relies on Bishop–Gromov monotonicity, see Remark 2.12, and Theorem 2.21.

In the following corollary we observe that for an arbitrary manifold with nonnegative Ricci curvature and Euclidean volume growth the isoperimetric profile is strictly increasing. This should be compared with [75, Theorem 3.3] where the author shows that on every complete noncompact Riemannian manifold with strictly positive sectional curvature the isoperimetric profile is strictly increasing.

#### Corollary 3.8

Let $(M^n,g)$ be a noncompact Riemannian manifold with $\mathrm {Ric}\ge 0$ and $\mathrm {AVR}(M^n,g)>0$. Then the isoperimetric profile $I_{(M^n,g)}$ is strictly increasing.

#### Proof

Since $I_{(M^n,g)}$ is concave due to Theorem 1.4, we have that $I'_+$ is nonincreasing. Moreover, since we have the asymptotic relation in (3.20) and $\mathrm {AVR}(M^n,g)>0$, we conclude that $I'_+\ge 0$. Hence, if for some $v_0>0$ we have $I'_+(v_0)=0$, therefore $I'_+(v)=0$ for every $v\ge v_0$, which is in contradiction with (3.20). In conclusion $I'_+>0$ everywhere, and hence *I* is strictly increasing. $\square$

## Main isoperimetric existence results

In this section we prove the main existence result Theorem 1.1 and the consequent Theorem 1.2 and Theorem 1.3.

### Proof of Theorem 1.1

Let us denote for simplicity $\theta :=\mathrm {AVR}(M^n,g)$. We divide the proof in two steps. The first step is the following Lemma in which we prove that, under the hypotheses of Theorem 1.1, for large volumes *V* any minimizing sequence of bounded finite perimeter sets of volume *V* can lose at most one piece at infinity along *N*.

#### Lemma 4.1

Let $k\ge 0$ be a natural number, and let $(M^n,g)=({\mathbb {R}}^k\times N^{n-k},g_{{\mathbb {R}}^k}+ g_N)$ be a complete noncollapsed Riemannian manifold with $\mathrm {Ric}\ge 0$. Assume that *M* satisfies the hypothesis of Theorem 1.1. Then there exists ${\overline{V}}>0$ such that the following holds.

For every $V\ge {\overline{V}}$, let $\{\Omega _i\}_{i\in {\mathbb {N}}}$ be a minimizing sequence for the volume *V* of bounded finite perimeter sets. Let $p_{i,j}:=(t_{i,j},x_{i,j})\in {\mathbb {R}}^k\times N$ be the points for which Theorem 2.16 applied to $\{\Omega _i\}_{i\in {\mathbb {N}}}$ holds, using the same notation therein, and for a fixed $x_0\in N$ let

4.1 $$\begin{aligned} \begin{aligned} J_1:=&\{j\in [1, L+1):\sup _i {\mathsf {d}}_N(x_0,x_{i,j})<+\infty \}, \\ J_2:=&\{j\in [1,L +1):\limsup _i {\mathsf {d}}_N(x_0,x_{i,j})= +\infty \}. \end{aligned} \end{aligned}$$

Then for every $j\in J_2$ we have $\lim _i {{\,\mathrm{vol}\,}}(\Omega _{i,j}^d) > V/2$. In particular, $J_2$ consists of at most one element for any $V \ge {\overline{V}}$.

#### Proof

Let us observe first that (3.21) implies that there exists ${\widehat{V}}>0$ such that

$$\begin{aligned} \begin{aligned} I'_{+}(v) < \frac{n}{n-1} (n-1)(\omega _n(\theta + \varepsilon /2))^{1/n} v^{-1/n}&\qquad \forall v \ge {\widehat{V}}, \\ \end{aligned} \end{aligned}$$

where the constant $\varepsilon$ is the one provided by the hypothesis in Theorem 1.1 . In particular

$$\begin{aligned} \begin{aligned} I(V)-I(V-\delta )&< \int _{V-\delta }^V \frac{n}{n-1} (n-1)(\omega _n(\theta + \varepsilon /2))^{1/n} v^{-1/n} \,\mathrm dv \\&\le n(\omega _n(\theta + \varepsilon /2))^{1/n} \frac{\delta }{(V-\delta )^{1/n}}, \end{aligned} \end{aligned}$$

whenever $V>V-\delta \ge {\widehat{V}}$. Also, we have that $\delta /(V-\delta )^{1/n} \le \delta ^{1-1/n}$ if $\delta \le V/2$. Therefore

4.2 $$\begin{aligned} I(V)-I(V-\delta ) < n(\omega _n(\theta + \varepsilon /2))^{1/n}\delta ^{1-1/n}, \end{aligned}$$

whenever $$V>V-\delta \ge {\widehat{V}}$$ and $$\delta \le V/2$$.

So we choose ${\overline{V}}:=2{\widehat{V}}$. Let $V\ge {\overline{V}}$ and $\{\Omega _i\}_{i\in {\mathbb {N}}}$ be an arbitrary minimizing sequence of bounded finite perimeter sets of volume *V*. Let $\Omega _{i,j}^d$ be defined as in Theorem 2.16, and we adopt the notation therein. Suppose by contradiction that ${\mathfrak {m}}_j(Z_j)=\lim _i {{\,\mathrm{vol}\,}}( \Omega _{i,j}^d) \le V/2$ for some $j\in J_2$. From Theorem 2.16 we have

$$\begin{aligned} I(V) = P_{X_j}(Z_j) + P(\Omega ) + \sum _{\ell \ne j} P_{X_\ell }(Z_\ell ) = P_{X_j}(Z_j)+ I({{\,\mathrm{vol}\,}}(\Omega )) + \sum _{\ell \ne j} I_{X_\ell }({\mathfrak {m}}_\ell (Z_\ell )) . \end{aligned}$$

By Theorem 2.21, by hypothesis, and by [13, Proposition 3.2] we then get

$$\begin{aligned} \begin{aligned} I(V)&\ge n(\omega _n(\theta + \varepsilon ))^{1/n}{\mathfrak {m}}_j(Z_j)^{(n-1)/n} + I({{\,\mathrm{vol}\,}}(\Omega )) + \sum _{\ell \ne j} I_{X_\ell }({\mathfrak {m}}_\ell (Z_\ell )) \\&\ge n(\omega _n(\theta + \varepsilon ))^{1/n}{\mathfrak {m}}_j(Z_j)^{(n-1)/n} + I \left( {{\,\mathrm{vol}\,}}(\Omega ) + \sum _{\ell \ne j} {\mathfrak {m}}_\ell (Z_\ell ) \right) \\&= n(\omega _n(\theta + \varepsilon ))^{1/n}{\mathfrak {m}}_j(Z_j)^{(n-1)/n} + I\left( V - {\mathfrak {m}}_j(Z_j) \right) . \end{aligned} \end{aligned}$$

By the absurd hypothesis we can take $\delta = {\mathfrak {m}}_j(Z_j)$ in (4.2) contradicting the above estimate, and thus completing the proof of the claim. $\square$

Let us now conclude the proof of Theorem 1.1 by exploiting the previous Lemma. Let us fix $p:=(0,x_0)\in {\mathbb {R}}^k\times N$. We now show that there exists $V_0\ge {{\overline{V}}}$ such that for every $V\ge V_0$ there exists an isoperimetric region of volume *V*, thus concluding the proof of the Theorem.

To reach the latter conclusion we start proving that there exists $V_0\ge {{\overline{V}}}$ such that the following improvement of Lemma 4.1 holds. Let $V\ge V_0$, and let $\{\Omega _i\}_{i\in {\mathbb {N}}}$ be a minimizing sequence for the volume *V* of bounded finite perimeter sets. Let $p_{i,j}:=(t_{i,j},x_{i,j})\in {\mathbb {R}}^k\times N$ be the points for which Theorem 2.16 applied to $\{\Omega _i\}_{i\in {\mathbb {N}}}$ holds, using the same notation therein, and define

$$\begin{aligned} \begin{aligned} J_1:=&\{j\in [1, L+1):\sup _i {\mathsf {d}}_N(x_0,x_{i,j})<+\infty \}, \\ J_2:=&\{j\in [1, L+1):\limsup _i {\mathsf {d}}_N(x_0,x_{i,j})= +\infty \}, \end{aligned} \end{aligned}$$

We claim that $J_2$ is actually empty.

Let us prove the previous claim by contradiction, after having provided the precise value of $V_0$. Let $\varepsilon$ be the constant given by the hypothesis of Theorem 1.1. Let us first consider the case $\theta >0$. Let us define

$$\begin{aligned} \varepsilon ^*:=(1+ \varepsilon /\theta )^{1/n}-1. \end{aligned}$$

Let $V_0\ge {{\overline{V}}}$ be big enough, where ${{\overline{V}}}$ is provided by Lemma 4.1, such that

4.3 $$\begin{aligned} I(V)\le n(\omega _n\theta )^{1/n}V^{(n-1)/n}\left( 1+2^{-1}\varepsilon ^*(1/3)^{(n-1)/n}\right) , \qquad \text {for all} V\ge V_0. \end{aligned}$$

Such a value of $V_0$ exists thanks to Corollary 3.6. From now on, we fix $V\ge V_0$ and we fix a minimizing sequence $\{\Omega _i\}_{i\in {\mathbb {N}}}$ of bounded finite perimeter sets of volume *V* for which, by contradiction, there exists exactly one $j_0\in J_2$, by Lemma 4.1. From (2.8) and the hypothesis we have

4.4 $$\begin{aligned} \frac{P_{X_{j_0}}(Z_{j_0})}{{\mathfrak {m}}_{j_0}(Z_{j_0})^{(n-1)/n}}\ge n\omega _n^{1/n}\mathrm {AVR}(X_{j_0},{\mathsf {d}}_{j_0},{\mathfrak {m}}_{j_0})^{1/n}\ge n\omega _n^{1/n}(\theta + \varepsilon )^{1/n}. \end{aligned}$$

From Lemma 4.1 and from item (iii) of Theorem 2.16 we have

4.5 $$\begin{aligned} {\mathfrak {m}}_{j_0}(Z_{j_0})=\lim _i{{\,\mathrm{vol}\,}}(\Omega _{i,{j_0}}^d)\ge V/3. \end{aligned}$$

For every $j\in J_1$, we have that $(M,{\mathsf {d}},p_{i,j},{{\,\mathrm{vol}\,}})\rightarrow _{i}(M,{\mathsf {d}},{{\overline{p}}}_j,{{\,\mathrm{vol}\,}})$ in the pmGH topology, for some ${{\overline{p}}}_j\in M$. Indeed, since $j\in J_1$ we have that, up to subsequences, $x_{i,j}\rightarrow _{i} {{\overline{x}}}_j\in N$. Hence it suffices to choose ${{\overline{p}}}_j:=(0,{{\overline{x}}}_j)$ thanks to the homogeneity in the factor ${\mathbb {R}}^k$. Hence, by the result in Theorem 2.16, we have that $\Omega _{i,j}\rightarrow _i Z_j$, where $Z_j$ is an isoperimetric region in *M*. Now, by exploiting item (iv) of Theorem 2.16, taking into account (4.4) and (4.5), exploiting also the isoperimetric inequality in Theorem 2.21, we finally get, recalling that $\varepsilon ^*:=(1+ \varepsilon /\theta )^{1/n}-1$, that

4.6 $$\begin{aligned} I(V)= & {} P(\Omega )+\sum _{j\in J_1}P(Z_j) + P_{X_{j_0}}(Z_{j_0}) \nonumber \\\ge & {} n(\omega _n\theta )^{1/n}\left( {{\,\mathrm{vol}\,}}(\Omega )^{(n-1)/n}+\sum _{j\in J_1}{{\,\mathrm{vol}\,}}(Z_j)^{(n-1)/n}+(1+ \varepsilon /\theta )^{1/n}{\mathfrak {m}}_{j_0}(Z_{j_0})^{(n-1)/n}\right) \nonumber \\\ge & {} n(\omega _n\theta )^{1/n}\left( \left( {{\,\mathrm{vol}\,}}(\Omega )+\sum _{j=1}^{L}{\mathfrak {m}}_j(Z_j)\right) ^{(n-1)/n}+\varepsilon ^*{\mathfrak {m}}_{j_0}(Z_{j_0})^{(n-1)/n}\right) \nonumber \\\ge & {} n(\omega _n\theta )^{1/n}V^{(n-1)/n}\left( 1+\varepsilon ^*(1/3)^{(n-1)/n}\right) , \end{aligned}$$

which is a contradiction with (4.3). Hence $J_2$ is empty.

Let us now consider the case $\theta =0$. From (3.20) we have that there exists $V_0\ge {{\overline{V}}}$ such that for all $V\ge V_0$ we have

4.7 $$\begin{aligned} I(V)\le n(\omega _n2^{-1}(1/3)^{(n-1)/n} \varepsilon )^{1/n}V^{(n-1)/n}. \end{aligned}$$

Arguing similarly as before we have, for every $V\ge V_0$, and for every minimizing sequence $\{\Omega _i\}_{i\in {\mathbb {N}}}$ of bounded sets of finite perimeter of volume *V*, by using the same notation as before,

4.8 $$\begin{aligned} \begin{aligned} I(V)&=P(\Omega )+\sum _{j\in J_1}P(Z_j)+P_{X_{j_0}}(Z_{j_0}) \ge P_{X_{j_0}}(Z_{j_0}) \\&\ge n(\omega _n \varepsilon )^{1/n}{\mathfrak {m}}_{j_0}(Z_{j_0})^{(n-1)/n}\ge n(\omega _n(1/3)^{(n-1)/n} \varepsilon )^{1/n}V^{(n-1)/n}, \end{aligned} \end{aligned}$$

which is a contradiction with (4.7), and thus $J_2$ is empty also in this case.

Let us now take $V_0\ge {{\overline{V}}}$ such that the previous claim about $J_2$ to be empty (for every minimizing sequence of bounded sets of finite perimeter of volume greater than $V_0$) holds. Let us now conclude that for every $V\ge V_0$ there exists an isoperimetric region of volume *V* in *M*. Indeed, let us take a minimizing sequence $\{\Omega _i\}_{i\in {\mathbb {N}}}$ of bounded finite perimeter sets of volume *V*. We have already shown that for every $j\in J_1$, and thus for all *j*’s since $J_2$ is empty as $V\ge V_0$, we have that $(M,{\mathsf {d}},p_{i,j},{{\,\mathrm{vol}\,}})\rightarrow _{i}(M,{\mathsf {d}},{{\overline{p}}}_j,{{\,\mathrm{vol}\,}})$ in the pmGH topology, for some ${{\overline{p}}}_j\in M$. From Proposition 2.17 we get that $Z_j$ is bounded for every *j* since it is an isoperimetric region in *M*. Hence, by properly translating $Z_j$ along the coordinate ${\mathbb {R}}^k$ into some $Z_j'$, we can define $\Omega ':=\sqcup _{j=1}^{L} Z'_j$, where the $Z_j'$s are mutually disjoint. Moreover, from (2.5) we get that ${{\,\mathrm{vol}\,}}(\Omega ')=V$. Hence, from (2.5), we conclude that

4.9 $$\begin{aligned} I(V)=P(\Omega )+\sum _{j=1}^{L} P(Z_j)=P(\Omega ')\ge I(V), \end{aligned}$$

from which $\Omega '$ is the sought isoperimetric region of volume *V*.

### Proof of Theorem 1.2

In order to complete the proof of Theorem 1.2 we will use Theorem 1.1 together with the following Lemma, that we state and prove in the general $\mathsf {RCD}$ setting. In item (i) of Lemma 4.2 we show that when the hypothesis of Theorem 1.2 holds, then the density of the points at distance one from the tips of the asymptotic cones of $M^n$ is uniformly greater than $\mathrm {AVR}(M^n,g)$. In item (ii) of Lemma 4.2 we show that when the previous conclusion on the density holds, then every pmGH limit at infinity has $\mathrm {AVR}$ uniformly greater than the $\mathrm {AVR}(M^n,g)$. The argument of the proof heavily relies on the cone splitting argument, cf. [32].

#### Lemma 4.2

Let $k\ge 0$ be a natural number, let $(Y,{\mathsf {d}}_Y,{\mathfrak {m}}_Y)$ be a metric measure space, and assume

$$\begin{aligned} (X:={\mathbb {R}}^k\times Y,{\mathsf {d}}:={\mathsf {d}}_{{\mathbb {R}}^k}\otimes {\mathsf {d}}_Y,{\mathfrak {m}}:={\mathfrak {m}}_{{\mathbb {R}}^k}\otimes {\mathfrak {m}}_Y), \end{aligned}$$

is an $\mathsf {RCD}(0,n)$ space. Assume

4.10 $$\begin{aligned} \mathrm {AVR}(X,{\mathsf {d}},{\mathfrak {m}})>0. \end{aligned}$$

Then the following two statements hold.

(i) If there exists no asymptotic cone of $(Y,{\mathsf {d}}_Y,{\mathfrak {m}}_Y)$ that splits a line, then there exists an ${\varepsilon }>0$ such that for every asymptotic cone
  $$\begin{aligned} ({\mathbb {R}}^k\times C,{\mathsf {d}}_{{\mathbb {R}}^k}\otimes {\mathsf {d}}_C,{\mathfrak {m}}_{{\mathbb {R}}^k}\otimes {\mathfrak {m}}_C,(0,v_\infty )), \end{aligned}$$
  of $(X,{\mathsf {d}},{\mathfrak {m}})$, and for every $p\in {\mathbb {R}}^k\times C$ with ${\mathsf {d}}_{{\mathbb {R}}^k}\otimes {\mathsf {d}}_C(p,{\mathbb {R}}^k\times \{v_\infty \})=1$, we have
  $$\begin{aligned}&\,\,\,\lim _{r\rightarrow 0}\frac{{\mathfrak {m}}_{{\mathbb {R}}^k}\otimes {\mathfrak {m}}_ C(B_r(p))}{\omega _nr^n}=:\vartheta [({\mathbb {R}}^k\times C,{\mathsf {d}}_{{\mathbb {R}}^k}\otimes {\mathsf {d}}_C,{\mathfrak {m}}_{{\mathbb {R}}^k}\otimes {\mathfrak {m}}_C,p)]\\&\quad \ge \mathrm {AVR}(X,{\mathsf {d}},{\mathfrak {m}})+{\varepsilon }. \end{aligned}$$
(ii) Assume that there exists $\alpha > 0$ such that
  $$\begin{aligned} \lim _{r\rightarrow 0}\frac{{\mathfrak {m}}_{{\mathbb {R}}^k}\otimes {\mathfrak {m}}_ C(B_r(p))}{\omega _nr^n}\ge \alpha , \end{aligned}$$
  for every asymptotic cone
  $$\begin{aligned} ({\mathbb {R}}^k\times C,{\mathsf {d}}_{{\mathbb {R}}^k}\otimes {\mathsf {d}}_C,{\mathfrak {m}}_{{\mathbb {R}}^k}\otimes {\mathfrak {m}}_C,(0,v_\infty )), \end{aligned}$$
  of $(X,{\mathsf {d}},{\mathfrak {m}})$ and for every $p\in {\mathbb {R}}^k\times C$ with ${\mathsf {d}}_{{\mathbb {R}}^k}\otimes {\mathsf {d}}_C(p,\mathbb R^k\times \{v_\infty \})=1$. Fix $x_0:=(0,y_0) \in {\mathbb {R}}^k\times Y$. Fix $\{x_i:=(t_i,y_i)\}_{i\ge 1}\subset X$ a sequence with ${\mathsf {d}}_Y(y_i,y_0)\rightarrow _i +\infty$, and let $X_{\infty }$ be a pmGH limit of a subsequence of $(X,{\mathsf {d}},{\mathfrak {m}},x_i)$, namely
  $$\begin{aligned} (X,{\mathsf {d}},{\mathfrak {m}},x_i)\rightarrow _i(X_{\infty },{\mathsf {d}}_\infty ,{\mathfrak {m}}_\infty ,x_\infty ), \end{aligned}$$
  in the pmGH sense up to a subsequence. Hence, $\mathrm {AVR}(X_{\infty },{\mathsf {d}}_\infty ,{\mathfrak {m}}_\infty )\ge \alpha$.

#### Proof

In the proof for simplicity we denote $\theta _X:=\mathrm {AVR}(X,{\mathsf {d}},{\mathfrak {m}})$. Inductively using [47, Theorem 7.4], one deduces that $(Y,{\mathsf {d}}_Y,{\mathfrak {m}}_Y)$ is an $\mathsf {RCD}(0,n-k)$ space. Moreover, one can check that $\mathrm{AVR}(Y,{\mathsf {d}}_Y,{\mathfrak {m}}_Y)>0$ as well.

Let us first prove item (i). Let us assume by contradiction that there exist, for every $i\in {\mathbb {N}}$, asymptotic cones $(C_i',{\mathsf {d}}_i,{\mathfrak {m}}_i,(0,v_{\infty ,i}))$ of $(X,{\mathsf {d}},{\mathfrak {m}})$, and points $p_i\in C_i'$ such that ${\mathsf {d}}_i(p_i,{\mathbb {R}}^k\times \{v_{\infty ,i}\})=1$ and

$$\begin{aligned} \vartheta [(C_i',{\mathsf {d}}_i,{\mathfrak {m}}_i,p_i)]\le \theta _X+1/i. \end{aligned}$$

Recall that $C_i'={\mathbb {R}}^k\times C_i$, for some cone $C_i$ with tip $v_{\infty ,i}$. Notice that by translating along ${\mathbb {R}}^k$ we may assume that the ${\mathbb {R}}^k$ coordinate of $p_i$ is 0. Let us first notice that the sequence $\{(C_i',{\mathsf {d}}_i,{\mathfrak {m}}_i,(0,v_{\infty ,i}))\}_{i\in {\mathbb {N}}}$ is precompact in the pmGH topology. Indeed, since any $(C_i',{\mathsf {d}}_i,{\mathfrak {m}}_i,(0,v_{\infty ,i}))$ is the pmGH limit of a rescaling of *X*, the $C_i'$’s are $\mathsf {RCD}(0,n)$ spaces with ${\mathfrak {m}}_i(B_1(0,v_{\infty ,i}))=\theta _X$ for every $i\in {\mathbb {N}}$, due to the volume convergence result in Theorem 2.6. Hence precompactness follows from Remark 2.7. Moreover, any limit of such a sequence of asymptotic cones must be an asymptotic cone of $(X,{\mathsf {d}},{\mathfrak {m}})$, due to the fact that the pmGH-topology, in our case, is metrizable, see [49, Theorem 3.15]. Indeed, by exploiting the latter information, a diagonal argument implies that any pmGH limit point of the sequence $\{(C_i',{\mathsf {d}}_i,{\mathfrak {m}}_i,(0,v_{\infty ,i}))\}_{i \in {\mathbb {N}}}$ is also an asymptotic cone of *X*.

Hence, let us take $(C',{\mathsf {d}}_{C'},{\mathfrak {m}}_{C'},(0,v_{\infty }))$ an asymptotic cone of $(X,{\mathsf {d}},{\mathfrak {m}})$, that is also a pmGH limit of the sequence $\{(C_i',{\mathsf {d}}_i,{\mathfrak {m}}_i,(0,v_{\infty ,i}))\}_{i\in {\mathbb {N}}}$. In a proper realization of such a pmGH limit, we can assume that, up to further subsequences, $p_i\rightarrow p\in C'$, and since ${\mathsf {d}}_i(p_i,{\mathbb {R}}^k\times \{v_{\infty ,i}\})=1$, we get by continuity of the distance that ${\mathsf {d}}(p,{\mathbb {R}}^{k}\times \{v_{\infty }\})=1$, and hence $p\notin {\mathbb {R}}^k\times \{ v_{\infty }\}$. Since $(0,v_{\infty })$ is one tip of the asymptotic cone $C'$, from the volume convergence result in Theorem 2.6, we conclude that

4.11 $$\begin{aligned} {\mathfrak {m}}_{C'}(B_r(0,v_{\infty }))=\theta _X\omega _nr^n, \end{aligned}$$

for every $r>0$. By an immediate consequence of Bishop–Gromov volume comparison and the volume convergence we also have that

4.12 $$\begin{aligned} \vartheta [(C',{\mathsf {d}}_{C'},{\mathfrak {m}}_{C'},p)]\ge \theta _X. \end{aligned}$$

Moreover, since the density $\vartheta$ is lower semicontinuous with respect to the pmGH convergence, see [39, Lemma 2.2 (i)], we also conclude that

4.13 $$\begin{aligned} \vartheta [(C',{\mathsf {d}}_{C'},{\mathfrak {m}}_{C'},p)]\le \liminf _{i\rightarrow +\infty }\vartheta [(C_i',{\mathsf {d}}_i,{\mathfrak {m}}_i,p_i)]\le \theta _X. \end{aligned}$$

Hence, from (4.12) and (4.13), we deduce that

4.14 $$\begin{aligned} \lim _{r\rightarrow 0}\frac{{\mathfrak {m}}_{C'}(B_r(p))}{\omega _nr^n}=:\vartheta [(C',{\mathsf {d}}_{C'},{\mathfrak {m}}_{C'},p)]=\theta _X. \end{aligned}$$

Since *p* and $(0,v_{\infty })$ are at finite distance, from (4.11), a simple argument involving the triangle inequality, and the monotonicity of Bishop–Gromov ratios (see Remark 2.12) we also deduce that

4.15 $$\begin{aligned} \lim _{r\rightarrow +\infty }\frac{{\mathfrak {m}}_{C'}(B_r(p))}{\omega _nr^n}=\theta _X. \end{aligned}$$

From (4.14), (4.15), and the monotonicity of the Bishop–Gromov ratios, see Remark 2.12, we deduce that ${\mathfrak {m}}_{C'}(B_r(p))=\theta _X\omega _nr^n$ for every $r>0$. Hence, from the result in [38, Theorem 1.1], we conclude that $C'$ is a metric cone with tip $p\notin {\mathbb {R}}^k\times \{ v_{\infty }\}$. Hence $C'={\mathbb {R}}^k\times C$ is also metric cone over a tip that is not in ${\mathbb {R}}^k\times \{v_\infty \}$, and then from [12, Proposition 1.18] we get that *C* splits a line. But this is not possible, since *C* is an asymptotic cone of *Y* that by hypothesis does not split a line, thus giving the sought contradiction.

Let us now prove item (ii). Since $(X,{\mathsf {d}},{\mathfrak {m}})$ is an $\mathsf {RCD}(0,n)$ space we conclude by stability that also $(X_\infty ,{\mathsf {d}}_\infty ,{\mathfrak {m}}_\infty )$ is an $\mathsf {RCD}(0,n)$ space. Let us denote ${\widetilde{x}}_{i}:=(0,y_i)$ and observe that $(X,{\mathsf {d}},{\mathfrak {m}},x_i)$ is isomorphic to $(X,{\mathsf {d}},{\mathfrak {m}},{\widetilde{x}}_i)$. Then we may suppose that the pmGH limit $(X_\infty ,{\mathsf {d}}_\infty ,{\mathfrak {m}}_\infty ,x_\infty )$ is obtained through (a sub)sequence of $(X,{\mathsf {d}},{\mathfrak {m}},{\widetilde{x}}_i)$.

Since we have that ${\mathsf {d}}_Y(y_i,y_0)\rightarrow _i +\infty$, if we set $\rho _i:={\mathsf {d}}(x_0,{\widetilde{x}}_i)$, we get that, up to subsequences in *i*, $(X,\rho _i^{-1}{\mathsf {d}},\rho _i^{-n}{\mathfrak {m}},x_0)$ pmGH-converges to an asymptotic cone

$(C',{\mathsf {d}}_{C'},{\mathfrak {m}}_{C'},(0,v_{\infty }))$. Notice that $C'={\mathbb {R}}^k\times C$ for some cone *C* with tip $v_\infty$. Moreover, in a realization of such a convergence, ${\widetilde{x}}_i\rightarrow _{i} p$ such that ${\mathsf {d}}_{C'}(p,{\mathbb {R}}^k\times \{z_\infty \})=1$ from the fact that both $x_0$ and ${\widetilde{x}}_i$ have zero component along ${\mathbb {R}}^k$. From the hypothesis we get that, for some $\alpha >0$, we have

$$\begin{aligned} \lim _{r\rightarrow 0}\frac{{\mathfrak {m}}_{C'}(B_r(p))}{\omega _nr^n}\ge \alpha . \end{aligned}$$

Hence, for every $\eta$ there exists some $\delta :=\delta (\eta )>0$ for which

4.16 $$\begin{aligned} \frac{{\mathfrak {m}}_{C'}(B_\delta (p))}{\omega _n\delta ^n} \ge \alpha -\eta . \end{aligned}$$

Since $(X,\rho _i^{-1}{\mathsf {d}},\rho _i^{-n}{\mathfrak {m}},{\widetilde{x}}_i)$ pmGH-converges to $(C',{\mathsf {d}}_{C'},{\mathfrak {m}}_{C'},p)$, the volume convergence result in Theorem 2.6 implies that

4.17 $$\begin{aligned} \lim _{i \rightarrow \infty } \frac{{\mathfrak {m}}(B_{\rho _i\delta }({\widetilde{x}}_i))}{\omega _n(\rho _i\delta )^n}=\frac{{\mathfrak {m}}_{C'}(B_\delta (p))}{\omega _n\delta ^n}. \end{aligned}$$

Hence, from (4.16) and (4.17) we get that

4.18 $$\begin{aligned} \frac{{\mathfrak {m}}(B_{\rho _i\delta }({\widetilde{x}}_i))}{\omega _n(\rho _i\delta )^n}\ge \alpha -2\eta , \qquad \text {for all} i\ge i_0(\eta ) \text {large enough}. \end{aligned}$$

Let us fix $R>0$. From the volume convergence result in Theorem 2.6 we have that

$$\begin{aligned} \lim _{i\rightarrow \infty } \frac{{\mathfrak {m}}(B_R({\widetilde{x}}_i))}{\omega _nR^n}= \frac{{\mathfrak {m}}_\infty (B_R(x_\infty ))}{\omega _nR^n}. \end{aligned}$$

By Bishop–Gromov volume comparison on *X* and (4.18) applied with $i\ge i_1(\eta ,R)$ large enough, we have

$$\begin{aligned} \frac{{\mathfrak {m}}_\infty (B_R(x_\infty ))}{\omega _nR^n}\ge \alpha -3\eta . \end{aligned}$$

Since $R,\eta >0$ are arbitrary we get the sought conclusion taking $R\rightarrow +\infty$ and then $\eta \rightarrow 0$. $\square$

#### Conclusion of the proof of Theorem 1.2

It is a direct consequence of Lemma 4.2, and Theorem 1.1. $\square$

### Further existence results

As a consequence of item (ii) of Lemma 4.2, we can derive further existence results on manifolds with nonnegative Ricci curvature and Euclidean volume growth. The next theorem states that if points located at distance 1 from the tips of every asymptotic cone of a manifold have density 1, then there exist isoperimetric regions of every volume. This is ultimately due to the fact that item (ii) of Lemma 4.2 implies that the essentially relevant pGH limits at infinity are ${\mathbb {R}}^n$, allowing us to conclude by the main existence result in [13].

#### Theorem 4.3

Let $k\ge 0$ be a natural number, and let $(M^n,g)=({\mathbb {R}}^k\times N^{n-k},g_{{\mathbb {R}}^k}+ g_N)$ be a complete Riemannian manifold such that $\mathrm {Ric}\ge 0$ and $\mathrm {AVR}(M^n,g)>0$. Assume that for every asymptotic cone $({\mathbb {R}}^k\times C,(0,v_\infty ))$ of *M* and every $p\in {\mathbb {R}}^k\times C$ with ${\mathsf {d}}_{{\mathbb {R}}^k}\otimes {\mathsf {d}}_C(p,{\mathbb {R}}^k\times \{z_\infty \})=1$, we have

4.19 $$\begin{aligned} \lim _{r\rightarrow 0}\frac{{\mathfrak {m}}_{{\mathbb {R}}^k}\otimes {\mathfrak {m}}_ C(B_r(p))}{\omega _nr^n}=:\vartheta [({\mathbb {R}}^k\times C,{\mathsf {d}}_{{\mathbb {R}}^k}\otimes {\mathsf {d}}_C,{\mathfrak {m}}_{{\mathbb {R}}^k}\otimes {\mathfrak {m}}_C, p)]=1. \end{aligned}$$

Then for every volume $V>0$ there exists an isoperimetric region of volume *V* on $M^n$.

#### Proof

We are in the setting of item (ii) of Lemma 4.2 with $\alpha =1$. From this we deduce that for any pmGH-limit $X_\infty$ at infinity as in the statement of item (ii) of Lemma 4.2 we have that $\mathrm {AVR}(X_\infty ,{\mathsf {d}}_\infty ,{\mathfrak {m}}_\infty )\ge 1$. But since $\mathrm {AVR}(X_\infty ,{\mathsf {d}}_\infty ,{\mathfrak {m}}_\infty )\le 1$ due to Bishop–Gromov comparison and the fact that ${\mathfrak {m}}_\infty ={\mathcal {H}}^n_{d_\infty }$ (cf. Theorem 2.6, and Remark 2.12), we have $\mathrm {AVR}(X_\infty ,{\mathsf {d}}_\infty ,{\mathfrak {m}}_\infty )= 1$. Therefore $(X_\infty ,{\mathsf {d}}_\infty ,{\mathfrak {m}}_\infty )=({\mathbb {R}}^n,{\mathsf {d}}_{{\mathbb {R}}^n},{\mathfrak {m}}_{{\mathbb {R}}^n})$ as a consequence of [39, Corollary 1.7] and the Bishop–Gromov comparison discussed in Remark 2.12.

Now if $k=0$, this implies that $(M^n, g)$ is GH-asymptotic to flat ${\mathbb {R}}^n$ (see [13, Definition 1.2]), and therefore on $M^n$ we have isoperimetric regions for every volume by [13, Theorem 5.2].

More generally, if $k>0$, we can still conclude that on $(M^n, g)$ we have isoperimetric regions of every volume $V>0$ arguing like at the end of the proof of Theorem 1.1. Indeed, pGH limits along sequences $(x_i,y_i)$ with $\{y_i\}_i$ bounded in *N* are isometric to $(M^n,g)$, and then possible mass of a minimizing sequence lost along these sequence can be obviously recovered as in the proof of Theorem 1.1. On the other hand we obtained that pGH limits obtained along sequences diverging along $N^{n-k}$ are flat ${\mathbb {R}}^n$, and then a minor modification in the proof of [13, Theorem 5.2] immediately shows that leak of the mass of a minimizing sequence along such sequences would lead to a contradiction of the minimality assumption on the sequence. $\square$

We record a straightforward consequence of the above statement.

#### Corollary 4.4

Let $(M^n,g)$ be a complete Riemannian manifold such that $\mathrm {Ric}\ge 0$ and $\mathrm {AVR}(M^n,g)>0$.

Assume that every asymptotic cone of $(M^n, g)$ is *smooth outside the tip*, i.e., it is of the form *C*(*Z*) where *Z* is a smooth closed manifold. Then for every volume $V>0$ there exists an isoperimetric region of volume *V* on $M^n$.

#### Proof

If every asymptotic cone of *M* is smooth outside the tip, then (4.19) is clearly satisfied, and thus the existence of isoperimetric regions follows. $\square$

Let us observe that, in the setting of Corollary 4.4, if $n=2$ then every asymptotic cone is (isometric to) a cone over a circle of some radius, which is obviously smooth outside the tip. Hence the latter statement, when specialized to $n=2$, recovers a particular case of the existence result of [74] on surfaces with nonnegative curvature.

### Proof of Theorem 1.3

As anticipated in the Introduction, for the proof of Corollary 1.3 we need the fact that manifolds with nonnegative sectional curvature have a unique asymptotic cone that splits if and only if the manifold splits. As explained in the Introduction, this is a standard result in the field, see [16, 54]. Nonetheless we give here, for the readers’ convenience, a proof in the only case we need for our aims, i.e., when $\mathrm {AVR}(M^n,g)>0$. We first prove an auxiliary lemma.

#### Lemma 4.5

Let $(X,{\mathsf {d}}_X)$ and $(Y,{\mathsf {d}}_Y)$ be compact metric spaces. If there exist maps $\Phi :X\rightarrow Y$ and $\Psi : Y \rightarrow X$ surjective and 1-Lipschitz, then $(X,{\mathsf {d}}_X)$ and $(Y,{\mathsf {d}}_Y)$ are isometric.

#### Proof

It is enough to check that $T:=\Psi \circ \Phi$ is an isometry, indeed from

$$\begin{aligned} {\mathsf {d}}_X(x,y) = {\mathsf {d}}_X(T(x),T(y)) \le {\mathsf {d}}_Y(\Phi (x),\Phi (y)) \le {\mathsf {d}}_X(x,y) \end{aligned}$$

we deduce that $\Phi$ is an isometry.

Fix distinct $x,y\in X$ and $\varepsilon \in (0,{\mathsf {d}}_X(x,y))$. Since *T* is 1-Lipschitz, it is enough to prove that ${\mathsf {d}}_X(T(x),T(y)) \ge {\mathsf {d}}_X(x,y)-\varepsilon$ and then let $\varepsilon \rightarrow 0$. Set $D:= {\mathsf {d}}_X(x,y)-\varepsilon /2$. Given an $\varepsilon /4$-dense set $S \subset X$, i.e., such that for any $z\in X$ there exists $s\in S$ with ${\mathsf {d}}_X(z,s)\le \varepsilon /4$, we define $N(S):= \#\{(s_1,s_2)\in S\times S\, : {\mathsf {d}}_X(s_1,s_2) \ge D\}$. Let $S_0$ be an $\varepsilon /4$-dense set that minimizes the function $N(\cdot )$ among $\varepsilon /4$-dense sets. Since *T* is 1-Lispchitz, then ${\mathsf {d}}_X(T(s_1),T(s_2))\le {\mathsf {d}}_X(s_1,s_2)$ for any $s_1,s_2\in S_0$, and since *T* is also surjective, then $T(S_0)$ is $\varepsilon /4$-dense, therefore $N(T(S_0))\ge N(S_0)$. This forces ${\mathsf {d}}_X(T(s_1),T(s_2))\ge D$ for any $s_1,s_2\in S_0$ with ${\mathsf {d}}_X(s_1,s_2)\ge D$. In particular, if we pick $s_1,s_2\in S_0$ such that ${\mathsf {d}}_X(x,s_1)\le \varepsilon /4$ and ${\mathsf {d}}_X(y,s_2)\le \varepsilon /4$ we have ${\mathsf {d}}_X(s_1,s_2)\ge -\varepsilon /2 + {\mathsf {d}}(x,y) =D$, and therefore, as *T* is 1-Lipschitz, we conclude

$$\begin{aligned} {\mathsf {d}}_X(T(x),T(y)) \ge {\mathsf {d}}_X(T(s_1), T(s_2)) - \varepsilon /2 \ge D-\varepsilon /2 = {\mathsf {d}}(x,y) - \varepsilon \, . \end{aligned}$$

$$\square $$

#### Theorem 4.6

If $(M^n,g)$ has nonnegative sectional curvature and Euclidean volume growth, then there exists a unique asymptotic cone $(C, {\mathsf {d}},{\mathcal {H}}^n)$. Moreover *C* splits if and only if *M* splits.

#### Proof

Let us begin by proving the uniqueness of asymptotic cones.

Thanks to Lemma 4.5, it is enough to show that, given two asymptotic cones $(C_1, {\mathsf {d}}_1, {\mathfrak {m}}_1)$ and $(C_2, {\mathsf {d}}_2, {\mathfrak {m}}_2)$, there exists a surjective 1-Lipschitz map $\Psi : {{\overline{B}}}_1(z_1) \rightarrow {{\overline{B}}}_1(z_2)$, where $z_1\in C_1$ and $z_2\in C_2$ are tip points.

Fix $p\in M$. Given $1<R_1<R_2$ we consider the set $E\subset B_{R_2}(p)$ of those points $x\in B_{R_2}(p)$ such that there exists a unique unit speed geodesic $\gamma _{p,x}:[0,{\mathsf {d}}(x,p)]\rightarrow M$ with $\gamma _{p,x}(0)=p$, $\gamma _{p,x}({\mathsf {d}}(x,p))=x$. It is well known [46, Lemma 3.96] that ${{\,\mathrm{vol}\,}}(B_{R_2}\setminus E)=0$, hence $E\subset B_{R_2}(p)$ is dense.

We then define $T: E\subset B_{R_2}(p) \rightarrow B_{R_1}(p)$ as $T(x):=\gamma _{p,x}({\mathsf {d}}(x,p)R_1/R_2)$. Toponogov’s theorem implies

4.20 $$\begin{aligned} {\mathsf {d}}(T(x), T(y)) R_1^{-1} \ge {\mathsf {d}}(x,y) R_2^{-1} \quad \text {for any} x,y\in E, \end{aligned}$$

in particular $T^{-1} : (T(E)\subset B_{R_1}(p), {\mathsf {d}}/R_1) \rightarrow (B_{R_2}(p), {\mathsf {d}}/R_2)$ is 1-Lipschitz and has dense image, hence it can be extended to a surjective 1-Lipschitz map $\Psi _{R_1,R_2}: (K_{R_1,R_2}, {\mathsf {d}}/R_1) \rightarrow ({{\overline{B}}}_{R_2}(p), {\mathsf {d}}/R_2)$, where $K_{R_1,R_2}:=\overline{T(E)}$.

Let us now consider two sequences $$r_i\rightarrow \infty $$ and $$s_i\rightarrow \infty $$ realizing $$(C_1, {\mathsf {d}}_1, {\mathfrak {m}}_1)$$ and $$(C_2, {\mathsf {d}}_2, {\mathfrak {m}}_2)$$, respectively. We assume without loss of generality that $$ s_i< r_i< s_{i+1}<r_{i+1}$$ for any $$i\ge 1$$. By the Gromov–Hausdorff convergence of the sequences of the rescaled space to the asymptotic cones, by a classical Ascoli–Arzelà-type argument we can pass to the limit the maps $$\Psi _{s_i,r_i}:((K_{s_i,r_i}, {\mathsf {d}}/s_i) \rightarrow ({{\overline{B}}}_{r_i}(p), {\mathsf {d}}/r_i)$$ and we derive the existence of a 1-Lipschitz surjective map

4.21 $$\begin{aligned} \Psi : K\subset {{\overline{B}}}_1(z_1) \rightarrow {{\overline{B}}}_1(z_2) \, , \end{aligned}$$

where $z_1\in C_1$ and $z_2\in C_2$ are tip points, and *K* is compact. Since $(M^n,g)$ has Euclidean volume growth, the volume convergence theorem, see Theorem 2.6, guarantees that the reference measures on the asymptotic cones is the Hausdorff measure (with respect to their own distance), and ${\mathcal {H}}^n((B_1(z_1)) = {\mathfrak {m}}_1(B_1(z_1)) = \omega _n\mathrm{AVR}(M^n,g) = {\mathfrak {m}}_2(B_1(z_2)) = {\mathcal {H}}^n(B_1(z_2))$, hence

$$\begin{aligned} {\mathcal {H}}^n((B_1(z_1))\setminus K)= & {} {\mathcal {H}}^n(B_1(z_1))-{\mathcal {H}}^n(K)\\= & {} {\mathcal {H}}^n(B_1(z_2)) -{\mathcal {H}}^n(K)\\\le & {} {\mathcal {H}}^n(B_1(z_2)) -{\mathcal {H}}^n(\Psi (K))\\= & {} 0, \end{aligned}$$

which yields $K={{\overline{B}}}_1(z_1)$ since *K* is closed. In particular (4.21) provides the sought map.

Let us now prove that the asymptotic cone $(C,{\mathsf {d}}, {\mathcal {H}}^n)$ splits if and only if *M* splits. In view of the splitting theorem it is enough to show the following implication

4.22 $$\begin{aligned} C \, \text {contains a line} \implies M \, \text {contains a line,} \end{aligned}$$

since the other one is readily verified by stability of the product structure under GH-convergence. Fix $p\in M$. It is enough to show that for any $0<\varepsilon <1/9$ there exist $p_1,p_2\in M$ satisfying ${\mathsf {d}}(p_1,p)={\mathsf {d}}(p_2,p) = 1/\varepsilon$ and

4.23 $$\begin{aligned} {\mathsf {d}}(p, \gamma _{p,p_1}(s)) + {\mathsf {d}}(p, \gamma _{p,p_2}(s)) \le {\mathsf {d}}(\gamma _{p,p_1}(s), \gamma _{p,p_2}(s)) + \varepsilon \quad \text {for any }s\in (0,1)\, , \end{aligned}$$

where $\gamma _{p,p_i}:[0,1]\rightarrow M$ is a minimizing constant speed geodesic such that $\gamma (0)=p$ and $\gamma (1)=p_i$, for $i=1,2$.

Indeed the curve $\gamma _\varepsilon :(-1/\varepsilon , 1/\varepsilon )\rightarrow M$, defined as

4.24 $$\begin{aligned} \gamma _\varepsilon :(-1/\varepsilon , 1/\varepsilon ) := {\left\{ \begin{array}{ll} \gamma _{p,p_1}(-t\varepsilon ) &{} \text {for }t\in (-1/\varepsilon , 0) \\ \gamma _{p,p_2}( t\varepsilon ) &{} \text {for }t\in (0, 1/\varepsilon )\, , \end{array}\right. } \end{aligned}$$

is 1-Lipschitz and satisfies $\gamma _\varepsilon (0)=p$. Hence, up to extracting a subsequence, $\gamma _\varepsilon \rightarrow \gamma$ locally uniformly, where $\gamma :{\mathbb {R}}\rightarrow M$. We claim that $\gamma$ is a line. To see this, we fix $t>0$ and we show that ${\mathsf {d}}(\gamma (t),\gamma (-t)) = 2t$. By (4.23) we have

$$\begin{aligned} \begin{aligned} 2t&= {\mathsf {d}}(p, \gamma _\varepsilon (-t)) + {\mathsf {d}}(p,\gamma _\varepsilon (t)) \le {\mathsf {d}}(\gamma _\varepsilon (-t), \gamma _\varepsilon (t)) + \varepsilon \le {\mathsf {d}}(\gamma _\varepsilon (-t),p ) + {\mathsf {d}}(p, \gamma _\varepsilon (t)) \\&\quad + \varepsilon \le 2t + \varepsilon . \end{aligned} \end{aligned}$$

Hence letting $\varepsilon \rightarrow 0$ implies $2t \le {\mathsf {d}}(\gamma (-t), \gamma (t)) \le 2t$ as claimed.

So we are left to prove (4.23). Given $0<\varepsilon <1/9$ we can find $R=R(\varepsilon ,n,p)>5/\varepsilon$ such that

4.25 $$\begin{aligned} {\mathsf {d}}_{GH}(B_R(p), B_R(z))\le \varepsilon ^2 R\, , \end{aligned}$$

where $z\in C(Z)$ is a tip. Since *C* splits a Euclidean factor, there exist $q_1,q_2\in B_R(p)$ satisfying

4.26 $$\begin{aligned} {\mathsf {d}}(p,q_1) = R, \quad {\mathsf {d}}(p,q_2) = R,\quad \text {and} \quad {\mathsf {d}}(p,q_1) + {\mathsf {d}}(p,q_2) \le {\mathsf {d}}(q_1,q_2) + \varepsilon ^2 R \, . \end{aligned}$$

Let $t\in (0,1)$ be such that ${\mathsf {d}}(p, \gamma _{p,q_1}(t))={\mathsf {d}}(p,\gamma _{p,q_2}(t))=1/\varepsilon$, where where $\gamma _{p,q_i}:[0,1]\rightarrow M$ is a minimizing constant speed geodesic such that $\gamma (0)=p$ and $\gamma (1)=q_i$, for $i=1,2$. We set $p_i:=\gamma _{p,q_i}(t)$ and check that

4.27 $$\begin{aligned} {\mathsf {d}}(p, \gamma _{p,q_1}(s)) + {\mathsf {d}}(p, \gamma _{p,q_2}(s)) \le {\mathsf {d}}(\gamma _{p,q_1}(s), \gamma _{p,q_2}(s)) + \varepsilon \quad \text {for any }s\in (0,t)\, , \end{aligned}$$

which amounts to our conclusion.

Toponogov’s theorem implies that ${\mathsf {d}}(\gamma _{p,q_1}(s), \gamma _{p,q_2}(s))\ge |{\tilde{\gamma }}_{{\tilde{p}},{\tilde{q}}_1}(s) - {\tilde{\gamma }}_{{\tilde{p}},{\tilde{q}}_2}(s)|$, where $({\tilde{p}}, {\tilde{q}}_1, {{\tilde{q}}}_2)$ is a comparison triangle in ${\mathbb {R}}^2$ corresponding to the triangle $(p,q_1,q_2)$, and ${\tilde{\gamma }}_{{{\tilde{p}}},{{\tilde{q}}}_i}$ is the constant speed Euclidean segment from ${{\tilde{p}}}$ to ${{\tilde{q}}}_i$, for $i=1,2$. The similarity of the triangles $({{\tilde{p}}}, {{\tilde{q}}}_1, {{\tilde{q}}}_2)$ and $({{\tilde{p}}}, {\tilde{\gamma }}_{{{\tilde{p}}},{{\tilde{q}}}_1}(s), {\tilde{\gamma }}_{{{\tilde{p}}},{{\tilde{q}}}_2}(s))$, together with (4.26), give

$$\begin{aligned} {\mathsf {d}}(\gamma _{p,q_1}(s), \gamma _{p,q_2}(s))&\ge |{\tilde{\gamma }}_{{{\tilde{p}}},{{\tilde{q}}}_1}(s) - {\tilde{\gamma }}_{{{\tilde{p}}},{{\tilde{q}}}_2}(s)| = |{{\tilde{q}}}_1 - {{\tilde{q}}}_2|\frac{ |{{\tilde{p}}} - {\tilde{\gamma }}_{{{\tilde{p}}},{{\tilde{q}}}_1}(s)|}{|{{\tilde{p}}} - {{\tilde{q}}}_1|} \\ {}&= {\mathsf {d}}(q_1,q_2)\frac{{\mathsf {d}}(p,\gamma _{p,q_1}(s)) + {\mathsf {d}}(p,\gamma _{p,q_2}(s))}{2R} \\ {}&\ge ({\mathsf {d}}(p,q_1) + {\mathsf {d}}(p,q_2) -\varepsilon ^2 R)\frac{{\mathsf {d}}(p,\gamma _{p,q_1}(s)) + {\mathsf {d}}(p,\gamma _{p,q_2}(s))}{2R} \\ {}&= (1-\frac{\varepsilon ^2}{2})( {\mathsf {d}}(p,\gamma _{p,q_1}(s)) + {\mathsf {d}}(p,\gamma _{p,q_2}(s))) \\ {}&\ge {\mathsf {d}}(p,\gamma _{p,q_1}(s)) + {\mathsf {d}}(p,\gamma _{p,q_2}(s)) -\varepsilon \, , \end{aligned}$$

where in the last step we used ${\mathsf {d}}(p,\gamma _{p,q_1}(s))= {\mathsf {d}}(p,\gamma _{p,q_2}(s))\le 1/\varepsilon$. $\square$

#### Remark 4.7

The statement of Theorem 4.6 holds in the class of Alexandrov spaces with nonnegative sectional curvature, as a refinement of the arguments above may show.

#### Conclusion of the proof of Theorem 1.3

The proof immediately follows from Theorem 4.6 and Theorem 1.2. $\square$

## Data Availability

Data sharing not applicable to this article as no datasets were generated or analysed during the current study.

## Footnotes

1 The first and the fourth author very recently provided, in a joint work with E. Pasqualetto and D. Semola [15], variational tools capable of estimating the second variation of the perimeter in nonsmooth spaces, giving an alternative proof of the concavity properties of the isoperimetric profile in our setting.
